# Dynamic Immune Responsiveness in Breast Cancer: Tumor Microenvironment Plasticity, Therapeutic Timing, and Clinical Translation

**DOI:** 10.3390/ijms27198854

**Published:** 2026-10-04

**Authors:** S. M. Nashir Udden, Monira Obaid, Laurentiu M. Pop, Raquibul Hannan

**Affiliations:** 1 Department of Radiation Oncology, University of Texas Southwestern Medical Center, 5323 Harry Hines Blvd., Dallas, TX 75390-9187, USA; 2 Department of Biological Sciences, University of Texas at Dallas, Richardson, TX 75080-3021, USA

**Keywords:** breast cancer, immunotherapy, immune responsiveness, tumor microenvironmental plasticity, therapeutic timing, immune checkpoint blockade, spatial biology, biomarkers, precision immunotherapy

## Abstract

Breast cancer immunotherapy has advanced substantially, yet durable clinical benefit remains limited to selected patient populations, reflecting heterogeneity in tumor–immune interactions across molecular subtypes, disease stages, and treatment contexts. This review frames immune responsiveness as a dynamic manifestation of tumor microenvironmental plasticity rather than a fixed tumor phenotype. We integrate mechanistic evidence showing that anti-tumor immunity is governed by interactions among malignant cells, immune populations, and cancer-associated fibroblasts. We emphasize therapeutic timing where treatment-induced changes in antigen availability, immune priming, stromal architecture, and cellular composition may create transient windows of immune susceptibility. Mechanisms of immune escape, including defective antigen presentation, T-cell dysfunction, suppressive myeloid populations, stromal exclusion, vascular abnormalities, and metabolic constraints, are evaluated as potential therapeutic targets. Emerging approaches incorporating single-cell and spatial profiling, circulating tumor DNA, multiomics, digital pathology, and artificial intelligence offer approaches for longitudinal characterization of evolving immune states, but their clinical utility in guiding immunotherapy remains largely unvalidated. We propose a precision-immunotherapy framework in which treatment selection and sequencing could be informed by these states. This framework complements established treatment approaches and defines testable hypotheses for prospective clinical studies.

## 1. Introduction

Immune checkpoint blockade (ICB) has transformed oncology by restoring antitumor immunity through inhibition of programmed cell death protein 1 (PD-1), programmed cell death ligand 1 (PD-L1), and cytotoxic T-lymphocyte-associated protein 4 (CTLA-4). Its success has shifted the field from a tumor-cell-centric view toward an integrated understanding of the cellular, molecular, and spatial interactions that regulate immunity within the tumor microenvironment (TME) [1,2,3,4,5]. Breast cancer, however, shows heterogeneous sensitivity to ICB (also known immune checkpoint inhibitor, ICI). Benefit is most established in triple-negative breast cancer (TNBC), particularly with neoadjuvant chemotherapy, whereas hormone receptor-positive/human epidermal growth factor receptor 2-negative (HR^+^/HER2^−^) tumors generally show less immune activation and HER2-positive outcomes vary by treatment context and biomarker status. Even in TNBC, durable responses are limited to a subset, indicating that “immune-inflamed,” “immune-desert,” and “immune-excluded” classifications cannot fully capture the dynamic determinants of response [5,6,7,8,9,10,11]. This heterogeneity highlights the need to define how tumor subtype, disease stage, and therapeutic context jointly shape immune susceptibility.

Although many breast cancers have lower tumor mutational burden (TMB) and fewer candidate neoantigens than highly immunogenic tumors, these features do not fully explain resistance. Single-cell and spatial analyses show that immune cells are often present even in nonresponding tumors. Resistance may instead arise from dysfunctional cellular interactions, spatial exclusion, stromal remodeling, suppressive myeloid populations, metabolic competition, abnormal vasculature, and progressive T-cell dysfunction [8,9]. Thus, immune-cell presence alone does not establish functional immunity; cellular state and access to malignant cells are equally important. Immune responsiveness is therefore increasingly viewed as a dynamic state shaped by tumor progression and therapy, rather than a fixed property of molecular subtype. Malignant cells interact with cancer-associated fibroblasts (CAFs), endothelial and immune populations, extracellular matrix (ECM), and soluble mediators to determine whether antitumor immunity is initiated, sustained, or suppressed. These compartments are plastic and can be remodeled by chemotherapy, endocrine therapy, radiotherapy, targeted therapy, antibody–drug conjugates (ADCs), DNA damage response inhibitors, and ICB [12,13,14,15,16,17]. Their reciprocal interactions can create transient windows of susceptibility or, alternatively, reinforce immune escape during treatment. Therapeutic timing adds another dimension. In the neoadjuvant setting, the intact primary tumor remains a source of tumor-associated antigens (TAAs) and immunogenic signals, while its native immune and stromal architecture can support antigen presentation, dendritic-cell activation, T-cell priming, clonal expansion, and systemic memory. In early-stage TNBC, adding checkpoint blockade to chemotherapy increases pathological complete response (pCR) and improves event-free outcomes in selected patients. Translational studies indicate that benefit reflects coordinated remodeling across immune and stromal compartments, supporting investigation of timing as a biological variable without isolating its causal contribution to clinical benefit [8,11,18]. This setting also permits paired tissue analyses that can connect early ecosystem changes with pathological and long-term clinical outcomes.

Single-cell and spatial technologies have resolved diverse lymphocyte states and shown that cellular location and neighborhood organization can matter as much as abundance. Cytotoxic lymphocytes may remain confined to stroma by CAF-rich barriers, ECM deposition, abnormal vasculature, or suppressive myeloid niches; conversely, tertiary lymphoid structures can support local antigen presentation and lymphocyte activation. Spatial architecture is therefore a key determinant of immune competence [7,19,20,21,22]. Integrating cellular phenotype with spatial context may better distinguish productive immune infiltration from ineffective or excluded responses.

This plasticity extends beyond conventional checkpoints. Innate sensing, type I interferon, transforming growth factor-β (TGF-β), vascular endothelial growth factor (VEGF), hypoxia, metabolism, immunogenic cell death, ferroptotic stress, and ECM remodeling jointly regulate immune recruitment and function. Treatments can consequently be both immunostimulatory and immunosuppressive: chemotherapy and radiotherapy may release antigens, yet persistent repair responses can promote fibrosis, myeloid suppression, and vascular dysfunction. Endocrine therapy, cyclin-dependent kinases 4 and 6 (CDK4/6) inhibition, ADCs, poly(ADP-ribose) polymerase (PARP) inhibition, and anti-angiogenic therapy also remodel the immune ecosystem. Treatment effects thus depend on how, when, and where the surrounding ecosystem is altered [14,15,16,17,23]. These competing effects help explain why mechanistically rational therapies may succeed only at particular doses, sequences, or disease stages. Effective antitumor immunity requires completion of the cancer-immunity cycle, from antigen release and dendritic-cell presentation through T-cell priming, trafficking, infiltration, tumor recognition, and sustained cytotoxicity [24]. Failure at any stage can limit response. Accordingly, no single biomarker, including PD-L1, tumor mutational burden, or tumor-infiltrating lymphocyte density, captures the complexity of response across breast cancer subtypes and settings. Immune competence instead reflects integrated cellular states, spatial organization, stromal architecture, metabolism, and treatment-induced temporal change (Figure 1) [7,9,13,24]. A recent editorial similarly emphasizes multimodal immune-biomarker development and the need to establish clinical utility; it is cited here as contemporary perspective rather than primary evidence of efficacy [25].

In this Review, we integrate clinical and mechanistic evidence on subtype-specific immunotherapy, spatial and temporal TME plasticity, neoadjuvant timing, immuno-radiobiology, resistance, rational combinations, and multidimensional biomarkers. We emphasize how mechanism and treatment context can inform patient selection and adaptive therapy.

### Literature Selection and Revision Search

This article is a narrative review rather than a systematic review or meta-analysis. Literature was identified using PubMed and Google and selected for its relevance to breast cancer immunotherapy, tumor–immune interactions, therapeutic timing, and clinical translation. Randomized breast-cancer trials and their follow-up reports were prioritized for efficacy claims, while human breast-cancer translational studies were prioritized for disease-specific biology. Preclinical studies and studies in other cancers were included for mechanistic interpretation, with their experimental or disease setting identified. Sources outside these topics were excluded. Reviews and editorials were used for context rather than as substitutes for primary trial evidence.

For the revision, targeted searches using PubMed and Google were completed on 25 September 2026 using trial names and terms related to breast cancer immunotherapy, neoadjuvant timing, spatial immune organization, radiotherapy, and biomarkers. These searches included checks of primary research articles and FDA notices. No publication-year restriction was applied to the revision searches. These checks supplemented the original literature selection and did not constitute exhaustive retrieval, independent duplicate screening, or formal risk-of-bias assessment.

## 2. Evolution of Breast Cancer Immunotherapy

### 2.1. From an Immunologically Cold Tumor to a Dynamic Immune Ecosystem

ICB transformed cancer treatment and redirected research toward the regulation of antitumor immunity. In breast cancer, early PD-1/PD-L1 inhibitor studies showed modest activity, particularly in heavily pretreated metastatic disease, whereas subsequent trials established benefit in defined TNBC settings [3,10,11,18]. The limited early responses reinforced the view of breast cancer as relatively immunologically cold, with lower TMB, fewer candidate neoantigens, and less endogenous immune activation than highly immunogenic tumors [1,2,4,26]. Single-cell and spatial studies in breast cancer have since shown why baseline lymphocyte abundance alone is insufficient to characterize the immune microenvironment: cellular states, spatial organization, and interactions also matter [6,7,8]. These findings support the dynamic framework introduced above. Table 1 summarizes the clinical trial evidence, and Section 4 examines its application across breast cancer subtypes and disease settings. Dendritic cells (DCs), natural killer (NK) cells, and other immune populations are discussed in Section 3, while radiotherapy (RT) and DNA damage response (DDR) interventions are considered in Section 6 and Section 8.

### 2.2. A New Paradigm for Breast Cancer Immunotherapy

The practical implication of this framework is to investigate which immune barriers can be modified, in which patients, and at which treatment stage. Mechanistically rational combinations are discussed in Section 8, while Section 9 separates established biomarkers from exploratory tools. This organization links the biology to testable clinical questions without treating ecosystem profiling as an already validated treatment-selection method.

## 3. Dynamic Immune Ecosystem of Breast Cancer

### 3.1. The TME as a Dynamic Biological System

The TME is increasingly recognized as a dynamic and adaptive ecosystem that evolves throughout tumor initiation, progression, metastasis, and therapeutic intervention. Rather than serving as a passive scaffold surrounding malignant epithelial cells, the breast cancer TME comprises an interconnected network of tumor cells, stromal fibroblasts, endothelial cells, ECM, and diverse innate and adaptive immune populations. These compartments communicate through direct cell–cell interactions, cytokines, chemokines, metabolites, extracellular vesicles, and mechanical signals, collectively shaping tumor evolution and determining whether anti-tumor immunity is initiated, sustained, or suppressed [13,39,40,41].

The cellular composition and functional state of the TME are not fixed. Genetic instability, hypoxia, metabolic stress, endocrine signaling, chemotherapy, radiotherapy, targeted therapy, and ICB can alter cellular abundance, activation states, intercellular communication, and tissue organization. Accordingly, immune responsiveness is better understood as an emergent property of the evolving tumor ecosystem than as an intrinsic characteristic of the malignant cell alone [5,13,26,40,41]. This dynamic perspective refines the conventional classification of tumors as “immune-inflamed,” “immune-desert,” or “immune-excluded.” Although these categories remain useful descriptive frameworks, they do not necessarily represent permanent tumor phenotypes. Individual tumors can acquire or lose immune infiltration, alter stromal architecture, and undergo shifts in myeloid and lymphoid composition during progression or treatment. The same tumor may therefore occupy different immunological states at different points in its natural history or therapeutic course [6,7,8,26] (Figure 2). Immune-inflamed, immune-desert, and immune-excluded describe spatial patterns of immune-cell distribution. Functional suppression can occur within any of these patterns and is shown separately as a cross-cutting feature, not a fourth spatial state. TLS enrichment represents an additional feature of tissue organization. Different patterns and functional features may coexist within a tumor. An inflamed pattern alone does not establish responsiveness to immunotherapy [7,22].

### 3.2. Cellular Architecture of the Breast Cancer Microenvironment

The breast cancer TME contains multiple interacting cellular compartments, each contributing distinct functions to immune regulation and tumor behavior [6,7,13] (Table 2). Malignant epithelial cells are active participants in immune regulation. In addition to uncontrolled proliferation, tumor cells can alter antigen presentation, express inhibitory ligands, secrete immunomodulatory cytokines and chemokines, compete with immune cells for metabolic substrates, and evolve under immune and therapeutic selection. Intratumoral heterogeneity further enables distinct malignant subclones to interact differently with immune and stromal populations and to adapt to selective pressures during treatment [6,7,26].

CAFs constitute a major stromal compartment and are functionally heterogeneous rather than a uniform cell population. CAF subsets participate in ECM deposition and remodeling, collagen organization, angiogenesis, inflammatory signaling, and regulation of immune-cell trafficking. Some fibroblast states may support inflammatory responses, whereas others contribute to fibrotic barriers and immune exclusion through TGF-β, CXCL12, IL-6, and related signaling pathways. Importantly, current evidence supports a context-dependent model of CAF function rather than a simple division into uniformly tumor-promoting and tumor-suppressive populations [12,52].

Endothelial cells likewise have functions that extend beyond vascular perfusion. Tumor-associated blood vessels regulate oxygen and nutrient delivery, vascular permeability, leukocyte adhesion and extravasation, and the accessibility of immune cells to malignant tissue. Aberrant angiogenesis can produce structurally and functionally abnormal vessels that restrict effective lymphocyte trafficking. These observations provide a biological rationale for vascular normalization and other strategies aimed at improving immune-cell access to tumors [55,56].

### 3.3. Innate Immune Cells Shape the Immunological Landscape

Innate immune populations provide an important interface between tumor biology and adaptive immunity. Their functional plasticity can either support immune surveillance or establish immunosuppressive conditions during tumor progression [47,48]. Tumor-associated macrophages (TAMs) are among the dominant immune populations in many breast tumors. Rather than existing as discrete M1 and M2 phenotypes, macrophages occupy a continuum of states shaped by local cytokines, metabolic conditions, tissue damage, hypoxia, and tumor-derived signals. Immunosuppressive macrophage programs can promote angiogenesis, ECM remodeling, tumor-cell survival, and suppression of T-cell function through mediators including IL-10, TGF-β, VEGF, arginase-associated pathways, and other immunoregulatory signals [48,49]. DCs are essential for antigen capture, processing, and cross-presentation and therefore represent a critical bridge between innate sensing and adaptive antitumor immunity. Effective DC activation supports priming and expansion of tumor-reactive T cells, whereas dysfunctional or suppressive tumor environments can impair DC maturation and antigen presentation [46]. NK cells provide rapid recognition and elimination of transformed cells through mechanisms that are not dependent on conventional tumor-antigen recognition by T-cell receptors. Nevertheless, hypoxia, suppressive cytokines, metabolic stress, prostaglandins, adenosine, and inhibitory receptor signaling can impair NK-cell function in tumors. Restoration of NK-cell activity is therefore an important area of investigation in next-generation immunotherapy [47]. Additional innate populations, including neutrophils, myeloid-derived suppressor cells (MDSCs), mast cells, eosinophils, and innate lymphoid cells, can exert context-dependent effects on tumor progression and immune regulation. Their activities include modulation of inflammation, ECM remodeling, angiogenesis, antigen presentation, and adaptive immune responses, underscoring the complexity of innate immune regulation within the breast tumor ecosystem [48,50].

### 3.4. Adaptive Immunity Determines Durable Tumor Control

Adaptive immunity is central to durable tumor control following effective antigen presentation and immune activation. Cytotoxic CD8^+^ T cells remain major mediators of malignant-cell elimination, but single-cell studies demonstrate considerable heterogeneity among tumor-infiltrating lymphocytes. Distinct populations include effector, proliferating, tissue-resident memory, progenitor-exhausted, terminally exhausted, regulatory, and bystander T-cell states, each with different transcriptional programs and functional properties [8,42,44]. Progenitor-exhausted T cells characterized by T cell-specific transcription factor 1 (TCF1) expression and sustained proliferative potential are particularly important in checkpoint-mediated antitumor responses. These populations can expand following PD-1 blockade and contribute to the replenishment of effector cells, whereas terminally exhausted populations exhibit more limited functional plasticity. Thus, the presence of PD-1 expressing T cells alone does not establish that a tumor contains an immune compartment capable of sustained therapeutic reinvigoration [42,44]. CD4+ T-cell populations in breast cancer include helper and regulatory subsets with different functional properties. In human breast tumors, Gu-Trantien et al. identified CXCL13-producing follicular helper T cells within tertiary lymphoid structures and found that a follicular helper T-cell signature was associated with patient outcomes and response to preoperative chemotherapy [61]. Plitas et al. identified tumor-infiltrating regulatory T cells with a distinct transcriptional phenotype, including elevated CCR8 expression, in human breast cancer [62]. These findings support considering CD4+ T-cell identity and tissue organization alongside overall lymphocyte abundance, but do not establish these features as validated predictors of checkpoint-inhibitor benefit. B cells have also emerged as important components of antitumor immunity. In appropriate tissue contexts, B cells can contribute to antigen presentation, antibody production, cytokine signaling, and formation of organized tertiary lymphoid structures (TLSs). Across several tumor types, B-cell rich immune niches and TLSs have been associated with improved immunotherapy responses, although their predictive value and biological role in breast cancer remain areas of active investigation [19,20].

### 3.5. Spatial Organization Governs Immune Function

A major advance in breast cancer immunology has been recognition that immune-cell abundance alone provides an incomplete description of immune competence. Spatial organization, cellular neighborhoods, and the physical relationships among malignant, stromal, vascular, and immune populations can profoundly influence treatment response [6,7,21]. Single-cell and spatially resolved analyses of human breast cancers have identified organized stromal-immune niches and distinct tumor ecosystems associated with different clinical phenotypes. Cytotoxic lymphocytes may be abundant but concentrated in stromal regions rather than in direct proximity to malignant cells. CAF-rich regions, dense ECM, abnormal vasculature, and suppressive myeloid populations can collectively restrict effective immune-cell access to tumor nests [7,21,53]. Conversely, organized immune structures such as TLSs can provide localized environments for B-cell and T-cell interactions, antigen presentation, and lymphocyte activation. Evidence from multiple cancer types indicates that TLS formation and B-cell organization can be associated with improved checkpoint-blockade responses; in breast cancer, these relationships remain promising but require additional prospective validation before they can be considered established clinical biomarkers [19,20]. Thus, the functional organization of the tumor ecosystem may provide information that is not captured by the absolute number of infiltrating immune cells. Spatial immune architecture should therefore be considered a complementary component of future biomarker strategies rather than simply an extension of conventional TIL quantification [11,19,20,22,34] (Figure 3). In human TNBC, Gruosso and colleagues linked compartment-specific immune localization to distinct stromal and epithelial expression programs and prognosis. This provides direct breast-cancer evidence for spatial heterogeneity, but does not validate an assay for selecting ICB [22].

### 3.6. Temporal Plasticity During Therapy

Temporal plasticity is a defining feature of the breast cancer immune ecosystem. Anticancer therapies can produce simultaneous immunostimulatory and immunosuppressive effects, creating transient windows of altered immune susceptibility [3,13]. Chemotherapy can promote immunogenic cell death, increase tumor-antigen availability, and alter suppressive immune populations. Radiotherapy can induce DNA-damage-associated innate immune signaling, including cyclic GMP-AMP synthase-stimulator of interferon genes (cGAS-STING) activation, while also remodeling vascular, stromal, and inflammatory compartments. CDK4/6 inhibition has been shown preclinically to enhance antigen-presentation and interferon-related programs, whereas endocrine therapy, PARP inhibition, ADCs, and anti-angiogenic therapies can also influence immune composition through distinct mechanisms [4,14,17,63,64,65]. These beneficial changes may be counterbalanced by adaptive responses involving fibrosis, macrophage polarization, abnormal angiogenesis, metabolic adaptation, T-cell exhaustion, and other immunosuppressive pathways. Consequently, immune activation may be transient unless therapeutic strategies are designed to sustain favorable ecosystem states and prevent compensatory resistance [12,48,55]. This temporal dimension provides a biological rationale for therapeutic sequencing. Neoadjuvant therapy preserves an intact primary tumor that can continue to supply TAAs and inflammatory signals during treatment, potentially supporting antigen presentation and immune priming before surgical removal. However, the proposition that the intact primary tumor universally functions as an “immunological reservoir” should be viewed as a biologically supported model rather than an established determinant of benefit for every patient [66].

## 4. Clinical Evidence Across Breast Cancer Subtypes

### 4.1. Clinical Heterogeneity Reflects Immunological Diversity

The clinical activity of ICB varies substantially across breast cancer subtypes and treatment settings. TNBC, HR^+^/HER2^−^ disease, and HER2^+^ tumors differ in immune infiltration, genomic characteristics, stromal architecture, and treatment sensitivity. Consequently, breast cancer should be viewed not as a single immunological entity but as a spectrum of tumor ecosystems with distinct and potentially dynamic immune states [5,26]. Importantly, molecular subtype alone does not fully predict treatment response. Within each subtype, only subsets of patients derive substantial benefit from checkpoint blockade, emphasizing the importance of tumor-specific immune architecture, biomarker status, and treatment context [4].

### 4.2. Triple-Negative Breast Cancer: The Prototype for Breast Cancer Immunotherapy

TNBC currently provides the strongest clinical evidence for ICB in breast cancer. Compared with many HR^+^/HER2^−^ tumors, TNBC more frequently exhibits TIL infiltration, immune-related gene-expression programs, genomic instability, and PD-L1 expression, creating biological conditions that may be more permissive to immune activation. Nevertheless, these features are heterogeneous and do not guarantee durable treatment benefit [5,26].

### 4.3. Metastatic TNBC

Early studies of PD-1 or PD-L1 blockade as monotherapy demonstrated modest response rates in metastatic TNBC, with greater activity in selected patients, particularly in earlier treatment lines and PD-L1^+^ disease. Subsequent randomized trials established combination chemoimmunotherapy as an important therapeutic strategy. In KEYNOTE-355, pembrolizumab plus chemotherapy produced a significant overall-survival benefit in patients with metastatic TNBC whose tumors had a PD-L1 combined positive score (CPS) of at least 10 [10]. Atezolizumab also showed clinically meaningful activity when combined with nab-paclitaxel in IMpassion130, particularly in PD-L1^+^ tumors, providing an early demonstration that checkpoint blockade could improve outcomes in metastatic TNBC [27,28]. However, the subsequent IMpassion131 trial failed to demonstrate a progression-free or overall-survival benefit when atezolizumab was combined with solvent-based paclitaxel, underscoring the importance of treatment backbone, patient selection, and trial context [30]. These results illustrate that immunotherapy efficacy cannot be extrapolated simply from the activity of a checkpoint inhibitor as a single agent.

### 4.4. Early-Stage TNBC

The most substantial clinical advance in breast cancer immunotherapy has occurred in early-stage TNBC. In KEYNOTE-522, the addition of pembrolizumab to neoadjuvant chemotherapy followed by adjuvant pembrolizumab significantly increased pCR and improved event-free survival compared with chemotherapy alone [11,18,31]. Mature overall-survival analysis subsequently demonstrated a significant survival benefit, confirming that the perioperative strategy provides benefits beyond improvement in pCR alone [18]. Other neoadjuvant trials have produced complementary but not uniformly concordant results. IMpassion031 demonstrated a significant increase in pCR with neoadjuvant atezolizumab plus chemotherapy, whereas NeoTRIPaPDL1 did not demonstrate a significant pCR improvement with the addition of atezolizumab. Longer-term GeparNuevo analyses have also suggested that the biological effects of neoadjuvant immune priming may not be fully captured by pCR alone [32,34,35]. Collectively, these studies demonstrate regimen-specific outcomes; differences in agents, chemotherapy, populations, endpoints, and follow-up prevent causal attribution of cross-trial differences to timing. Translational studies further support ecosystem-level interpretation. Effective neoadjuvant treatment has been associated with changes in proliferating and progenitor T-cell populations, interferon-related signaling, DC activity, and other immune compartments, whereas resistant tumors may retain stromal and myeloid programs associated with immune exclusion [8,44]. PD-L1 testing is not required to select eligible patients with high-risk early-stage TNBC for perioperative pembrolizumab; this differs from advanced TNBC, where CPS ≥ 10 is an established selection criterion for pembrolizumab-containing first-line regimens [10,11,18]. Treatment benefit must also be weighed against harm in potentially curable patients. KEYNOTE-522 reported estimated 5-year OS of 86.6% versus 81.7%, an absolute difference of 4.9 percentage points [18]. Immune-mediated toxicities can include thyroid dysfunction, adrenal insufficiency, hypophysitis, hepatitis, colitis, and pneumonitis; some endocrinopathies require long-term replacement therapy. Toxicity can interrupt or permanently discontinue treatment, and rare events can be fatal. These risks support careful eligibility assessment, monitoring, and discussion of absolute benefit, comorbidity, and lasting treatment burden rather than escalation based solely on exploratory biomarkers [11,31].

### 4.5. Hormone Receptor-Positive/HER2-Negative Breast Cancer: Overcoming Immune Quiescence

HR^+^/HER2^−^ breast cancer generally demonstrates lower levels of immune infiltration and inflammatory signaling than TNBC, and the clinical role of ICB remains more selective. Nevertheless, HR^+^/HER2^−^ tumors are immunologically heterogeneous rather than uniformly “cold,” and subsets exhibit measurable immune activation and inflammatory gene-expression programs [5,21,26]. Endocrine signaling can influence cytokine networks, antigen presentation, macrophage behavior, and lymphocyte recruitment. In parallel, preclinical studies demonstrate that CDK4/6 inhibition can increase antigen presentation and interferon-associated signaling while altering regulatory T-cell biology, providing a mechanistic rationale for combination with checkpoint blockade [14]. In KEYNOTE-756 (n = 1278), pCR was 24.3% versus 15.6%, with an estimated treatment difference of 8.5 percentage points (95% CI, 4.2–12.8); EFS was immature in the reported analysis [36]. CheckMate 7FL also improved pCR (Table 1), but its survival evidence does not establish a routine indication [37]. These findings support further investigation in selected high-risk ER^+^/HER2^−^ disease; they do not establish benefit from endocrine therapy-ICB alone or justify routine checkpoint blockade in this population.

### 4.6. HER2-Positive Breast Cancer: Bridging Targeted Therapy and Immunity

HER2^+^ breast cancer provides a biologically distinctive interface between targeted therapy and immune regulation. HER2-directed monoclonal antibodies can recruit innate immune effector mechanisms, including NK-cell-mediated antibody-dependent cellular cytotoxicity (ADCC), in addition to inhibiting HER2 signaling. This provides a mechanistic rationale for combining HER2-targeted therapies with immunomodulation [67,68]. The clinical results of checkpoint blockade combined with HER2-directed therapy have, however, been heterogeneous and context-dependent. Higher TIL levels, PD-L1 expression, and other immune features may help define patient subsets more likely to benefit, but no single immune biomarker currently provides sufficiently robust predictive performance across HER2^+^ disease [5]. The continued development of HER2-directed ADCs, including trastuzumab deruxtecan, has further expanded interest in combining targeted cytotoxic delivery with immunotherapy. ADCs can produce potent tumor-cell killing and depending on the payload and mechanism of cell death, may increase antigen availability and alter the immune microenvironment. Whether these effects can be translated into durable clinical synergy with checkpoint blockade remains an active area of investigation [69,70,71,72,73].

The relevant trials illustrate the limits of extrapolation. PANACEA was a single-arm phase Ib/II study of pembrolizumab plus trastuzumab in trastuzumab-resistant advanced disease, with activity in a subset of PD-L1-positive tumors [71]. KATE2 did not significantly improve PFS in the overall randomized population when atezolizumab was added to trastuzumab emtansine [72]. In early HER2-positive disease, IMpassion050 did not improve pCR with atezolizumab added to chemotherapy and trastuzumab–pertuzumab [73]. Exploratory subgroup findings from these studies do not establish a general indication for ICB in HER2-positive breast cancer.

### 4.7. Rare Breast Cancer Subtypes

Less common breast cancer subtypes and molecularly defined populations may harbor distinct immune ecosystems, but clinical evidence for ICB remains comparatively limited. Inflammatory breast cancer can exhibit substantial inflammatory signaling and immune infiltration while simultaneously developing suppressive myeloid and stromal programs. Tumors with BRCA1/2 mutations or homologous-recombination deficiency may display increased genomic instability and altered DNA-damage signaling, providing a biological rationale for combining DDR inhibition with immunotherapy. These hypotheses remain under active investigation and should not be interpreted as established clinical indications [4,17]. Male breast cancer and hereditary breast cancer syndromes remain substantially underrepresented in clinical immunotherapy studies. Dedicated studies and more inclusive trial designs will be necessary to define the immune biology and therapeutic relevance of these populations [21,70].

### 4.8. Cross-Subtype Determinants of Immunotherapy Response

Despite substantial differences among molecular subtypes, several biological requirements appear to be shared across breast cancers. Effective anti-tumor immunity requires adequate antigen availability, functional antigen presentation, productive T-cell priming, efficient lymphocyte trafficking, and sustained effector function. Conversely, stromal fibrosis, suppressive myeloid populations, metabolic competition, abnormal vasculature, chronic inflammatory signaling, and progressive T-cell dysfunction can limit therapeutic efficacy across multiple disease contexts [8,12,42,48,55]. Importantly, these determinants evolve during therapy. Chemotherapy, radiotherapy, endocrine therapy, targeted agents, and ADCs can remodel immune composition and create transient periods of altered immune susceptibility. This provides a mechanistic rationale for treatment sequences designed around biological state rather than the simple concurrent addition of multiple agents [4,14,17,63,64]. Across breast cancer subtypes, clinical benefit depends on tumor immune context, treatment setting, and regimen design rather than molecular subtype alone. Selected phase III trials and their principal implications are summarized in Table 3.

## 5. Neoadjuvant Immunotherapy and Therapeutic Timing

### 5.1. Therapeutic Timing as a Testable Biological Variable

The clinical success of neoadjuvant ICB has reshaped understanding of immunotherapy in breast cancer. Although immune checkpoint inhibitors initially demonstrated modest activity in advanced breast cancer, clinically meaningful benefit has subsequently been established in selected patients with early-stage disease, particularly TNBC. These observations indicate that immunotherapy efficacy depends not only on the therapeutic agent and molecular subtype but also on the biological context in which treatment is delivered. Therapeutic timing should therefore be defined as a set of distinct, testable variables rather than as a single preoperative-versus-postoperative comparison. These variables include: (i) treatment before versus after surgery; (ii) concurrent versus sequential combinations; (iii) treatment relative to antigen release and dendritic-cell (DC) priming; (iv) intervention before or during adaptive resistance; (v) the interval and order between radiotherapy (RT) and ICB; and (vi) the timing of longitudinal tissue and blood collection. Each operates on a different biological timescale and should be analyzed separately [11,18,31,66]. Unlike postoperative adjuvant therapy, neoadjuvant treatment is delivered while the primary tumor remains intact. The tumor can therefore continue to provide TAAs, inflammatory signals, and damage-associated molecular patterns (DAMPs) generated through spontaneous or therapy-induced tumor-cell death. These signals may be captured by antigen-presenting cells (APCs) and contribute to T-cell priming and clonal expansion while the tumor and its associated immune microenvironment remain physically connected. This provides a compelling rationale for testing immunotherapy before surgery, but it should not be interpreted as evidence that retaining the primary tumor is uniformly beneficial. The same lesion can serve simultaneously as an antigen source and as a systemic immunosuppressive organ; the net effect likely depends on tumor burden, duration of exposure, metastatic risk, and the balance between productive priming and tumor-driven tolerance [63,78].

### 5.2. The Intact Tumor as an Immunological Reservoir

The intact primary tumor can supply antigen and preserve local antigen-presenting and lymphocyte interactions, but can also sustain suppressive myeloid and stromal programs [46,48,50,66]. Its presence is therefore not intrinsically beneficial. Preclinical evidence provides a rationale for testing immune priming before resection, while the competing effects of tumor progression and suppression argue against extending treatment intervals solely on mechanistic grounds [66]. Immunogenic cell death can increase antigen availability and provide danger signals that support priming; the underlying mechanisms are detailed in Section 6.2.

Preclinical models have provided important evidence that treatment administered before surgical removal of the primary tumor can generate broader systemic antitumor immunity than equivalent treatment administered after resection. Neoadjuvant immunotherapy has been associated with enhanced tumor-specific T-cell responses and improved control of distant disease in experimental models. These findings provide a mechanistic rationale for investigating treatment timing as a biological variable rather than treating it solely as a clinical scheduling decision [66]. Clinical translational studies similarly indicate that neoadjuvant checkpoint blockade can remodel multiple immune compartments. Changes in proliferating and stem-like T-cell populations, DC activity, interferon-related signaling, and other immune states have been observed during treatment. Importantly, however, these observations do not establish that all clinical benefit derives from immune-memory formation before surgery; the relative contributions of neoadjuvant and adjuvant treatment remain context-dependent [6,8].

### 5.3. Remodeling the TME During Neoadjuvant Therapy

One of the most important insights from neoadjuvant immunotherapy is that checkpoint blockade can influence multiple components of the TME rather than simply increasing cytotoxic T-cell activity. Single-cell studies of breast tumors treated with ICB have identified treatment-associated changes in malignant, immune, and stromal populations, highlighting the extensive cellular remodeling that accompanies therapeutic response [8]. Following treatment, changes in DC activation, T-cell proliferation and differentiation, myeloid-cell states, interferon signaling, and inflammatory communication can alter the balance between immune activation and immune suppression. Spatially organized immune niches may also evolve during treatment, potentially facilitating interactions among antigen-presenting cells, lymphocytes, and malignant cells [7,8,21]. These favorable changes may nevertheless be counterbalanced by compensatory mechanisms, including TGF-β signaling, VEGF-associated vascular dysfunction, macrophage reprogramming, stromal fibrosis, metabolic adaptation, and expression of alternative inhibitory receptors. Thus, the biological response to ICB is not uniformly progressive toward immune activation but represents a dynamic competition between stimulatory and suppressive processes [12,48,79,80].

### 5.4. What Neoadjuvant Trials Establish About Timing

The trial outcomes summarized in Section 4.4 and Table 1 establish the effects of specific treatment regimens within their respective study populations. Perioperative trials such as KEYNOTE-522 and IMpassion031 do not isolate the contributions of neoadjuvant and adjuvant ICB because they evaluated strategies incorporating both phases. GeparNuevo, by contrast, evaluated neoadjuvant durvalumab without adjuvant continuation. Although it informs the potential benefit of preoperative ICB, it does not establish the superiority of preoperative over postoperative administration because these schedules were not directly randomized. Comparisons among KEYNOTE-522, IMpassion031, NeoTRIP, GeparNuevo, and adjuvant A-BRAVE cannot isolate timing effects because their populations, agents, chemotherapy backbones, endpoints, and follow-up differ [11,18,32,33,34,38]. GeparNuevo’s exploratory window analyses provide a rationale for investigating immune priming but do not define an optimal treatment schedule. Direct randomized schedule comparisons, together with prespecified longitudinal measurements, are needed to determine the contributions of treatment timing and assess whether particular immune transitions mediate clinical benefit. Figure 4 illustrates candidate temporal changes rather than an established clinical schedule.

### 5.5. Establishment of Systemic Immune Memory

One proposed advantage of neoadjuvant immunotherapy is the opportunity to generate durable systemic immune surveillance while the primary tumor remains present. Effective immune activation can generate effector and memory T-cell populations that recognize tumor-associated antigens beyond the primary lesion. Tissue-resident memory populations can support local surveillance, while circulating memory populations may contribute to systemic immune protection [81,82]. In preclinical breast cancer models, direct comparisons of the same immunotherapy administered before or after tumor removal have supported a potential advantage of neoadjuvant treatment for systemic antitumor immunity and protection against metastatic disease [66]. These findings provide a mechanistic rationale for investigating treatment timing but do not establish equivalent benefits in human breast cancer. Whether neoadjuvant administration produces more durable immune memory or metastatic protection than postoperative administration in patients remains unresolved. The generation and maintenance of antitumor memory involve antigen presentation, costimulation, and interactions among antigen-presenting cells and lymphocyte populations [46,81,82]. Whether treatment strategies that preserve these interactions improve long-term disease control requires direct testing. Figure 4 presents a hypothetical framework for investigating temporal immune changes, rather than a validated schedule or a monitoring strategy with established clinical utility.

### 5.6. Why Timing Matters Beyond Surgery

The biological principles associated with therapeutic timing may extend beyond the neoadjuvant setting. Chemotherapy, radiotherapy, endocrine therapy, CDK4/6 inhibitors, PARP inhibitors, ADCs, anti-angiogenic therapies, and checkpoint inhibitors can each produce distinct and sometimes transient changes in antigen availability, inflammatory signaling, stromal architecture, vascular function, and immune-cell composition [12,14,15,17,55,64,65].

These observations require several timing questions to be separated explicitly. First, perioperative timing asks whether immune therapy is initiated before resection, after resection, or across both phases. Second, combination timing asks whether agents should be given concurrently or sequentially; concurrency may maximize mechanistic overlap but can also compound toxicity or expose newly activated lymphocytes to cytotoxic injury, whereas sequencing can create a priming interval but risks missing a transient window of susceptibility. Third, immune-event timing aligns treatment with antigen release, DC activation, and T-cell priming. A therapy that increases antigen availability may need to precede or overlap checkpoint blockade, but the optimal interval depends on the kinetics of cell death, antigen presentation, lymphocyte expansion, and trafficking. Fourth, resistance timing asks whether a second intervention should be delivered prophylactically or only when adaptive programs, such as alternative checkpoints, myeloid recruitment, TGF-β signaling, or stromal exclusion, emerge [12,14,17,55,64,65]. RT-ICB timing warrants separate consideration. RT can rapidly release antigen and DAMPs, activate innate sensing, and alter trafficking, but it can also cause lymphocyte depletion, recruit suppressive myeloid cells, induce TGF-β, and upregulate inhibitory pathways. ICB administered before RT may expand or reinvigorate tumor-reactive cells available to respond to released antigen; concurrent treatment may couple checkpoint relief to inflammatory antigen presentation; and ICB after RT may capitalize on newly primed clones. These approaches are not biologically equivalent. Their effects depend on RT dose, fractionation, target volume, lymphoid-organ exposure, and the interval between RT and ICB; therefore, “RT plus ICB” should not be treated as a single scheduling category. Biomarker timing is an independent design variable. Pretreatment sampling measures baseline state but cannot define treatment-induced transition. An early on-treatment sample can capture target engagement, antigen release, innate activation, and initial priming; an intermediate sample can identify clonal expansion, trafficking, and emergent adaptive resistance; and tissue at surgery can characterize residual disease and persistent suppressive niches. Serial blood sampling between tissue collections can track circulating tumor DNA, cytokines, immune-cell states, and T-cell clonotypes with lower procedural burden. Sampling windows should therefore be anchored to biological events and each treatment exposure, not merely to clinic visits, and analyzed as trajectories rather than isolated values. However, the concept of an optimal sequence remains investigational. Current clinical evidence is strongest for specific established regimens, such as perioperative pembrolizumab-based therapy in early TNBC, rather than for a general principle that one universal sequence should be applied across breast cancer subtypes [11,18,31]. These observations motivate schedule-specific trials; they do not establish a universal therapeutic window or justify changing an effective regimen on the basis of unvalidated immune measurements.

## 6. Immuno-Radiobiology as a Distinctive Mechanistic Focus

### 6.1. Radiotherapy Beyond Local Tumor Control

Radiotherapy (RT) has traditionally been regarded primarily as a local treatment that eradicates malignant cells through the induction of DNA damage. Increasing evidence, however, demonstrates that irradiation also modifies the tumor immune microenvironment, influencing innate immune sensing, antigen presentation, adaptive immunity, stromal and vascular compartments, and ECM organization. These effects provide the rationale for treating immuno-radiobiology as a distinctive mechanistic focus of this review, rather than implying that RT-ICB combinations already have a mature breast-cancer clinical evidence base. Unless otherwise specified, the mechanistic concepts below derive predominantly from preclinical studies and clinical experience in other tumor types and should be regarded as hypothesis-generating for breast cancer [65,83,84,85] (Figure 5). The immunological consequences of RT are strongly context-dependent. Radiation dose, fractionation, target volume, treatment timing, tumor biology, baseline immune composition, and concomitant systemic therapy can all influence whether irradiation produces predominantly immunostimulatory or immunosuppressive effects. Consequently, RT can create transient conditions favorable to antitumor immunity while simultaneously activating mechanisms that limit the durability of that response [65,84,85,86]. This biological complexity explains the focused discussion that follows; it does not establish clinical efficacy of RT-ICB in breast cancer.

### 6.2. Radiation-Induced Immunogenic Cell Death

One important mechanism through which RT may stimulate antitumor immunity is the induction of immunogenic cell death (ICD). ICD is characterized by cellular stress and death accompanied by the release or exposure of DAMPs, including calreticulin, adenosine triphosphate (ATP), and high-mobility group box 1 (HMGB1). These signals can promote uptake of dying tumor cells, DC activation, antigen presentation, and subsequent adaptive immune responses [15,63,87]. Following irradiation, tumor cells can undergo apoptosis, necrosis, mitotic catastrophe, senescence, and other forms of stress-associated cell death. The immunological consequences vary according to tumor type and treatment parameters, and not every form of radiation-induced cell death is necessarily immunogenic. Radiation dose and fractionation are important determinants of these effects. In this subsection, evidence concerning dose, fractionation, cGAS-STING signaling, and TREX1 is principally preclinical. Preclinical work indicates that very high single doses can induce regulatory mechanisms that attenuate radiation-associated innate immune signaling, whereas appropriately fractionated regimens may preserve or enhance immunogenic signaling. Importantly, the optimal immunological dose and fractionation schedule are not universal and remain an active area of investigation [64,86].

Innate Immune Activation Through the cGAS-STING Pathway. The cGAS-STING pathway represents an important molecular link between radiation-induced DNA damage and innate immune activation. Irradiation can generate cytoplasmic DNA through chromosome damage, micronucleus formation, and altered DNA repair. Cytoplasmic DNA can activate cGAS, leading to production of cyclic GMP-AMP (cGAMP), STING activation, downstream TBK1 and IRF3 signaling, and induction of type I interferons and inflammatory mediators [64,88,89]. These signals can promote DC activation, antigen presentation, recruitment of NK cells and cytotoxic T lymphocytes, and establishment of conditions favorable to adaptive antitumor immunity. Experimental evidence has established that intact DNA-sensing pathways are important contributors to radiation-induced immune responses [64,88,89]. However, radiation-induced innate immune activation is tightly regulated. Vanpouille-Box and colleagues demonstrated that radiation doses above approximately 12–18 Gy can induce the DNA exonuclease TREX1, which degrades cytosolic DNA and attenuates cGAS-STING signaling. In experimental models, repeated irradiation at doses below those inducing strong TREX1 expression enhanced interferon signaling, dendritic-cell recruitment, and CD8^+^ T-cell priming. These observations provide mechanistic support for the importance of radiation dose and fractionation when RT is being considered as an immunological intervention [86].

### 6.3. Mechanistic Remodeling: Adaptive Immunity, Stroma, and Vasculature

RT can alter both tumor-cell immunogenicity and the composition and function of immune populations within TME. Radiation-associated cellular stress can increase antigen availability and influence antigen-presentation machinery, thereby potentially enhancing recognition of irradiated tumor cells by adaptive immune cells. Dendritic-cell activation and migration can support priming of tumor-reactive T cells, while inflammatory signals generated after irradiation can alter lymphocyte trafficking and local immune-cell recruitment [64,65,84]. Radiation can also modify the phenotype and function of T cells, NK cells, macrophages, dendritic cells, and other immune populations. These effects are highly dependent on the radiation regimen and the surrounding tumor ecosystem. Whether RT reliably establishes durable tissue-resident and systemic immune memory in human breast cancer remains unresolved; Section 5.5 discusses the distinction between memory biology and clinical evidence [81,82]. B cells and TLSs may also participate in radiation-associated immune remodeling. However, the extent to which RT consistently induces clinically meaningful TLS formation in breast cancer remains uncertain. Thus, TLS remodeling should be regarded as a promising area of investigation rather than an established consequence of breast irradiation [19,20]. RT affects non-immune components of the tumor ecosystem as well as malignant and immune cells. Irradiation can alter endothelial-cell activation, vascular permeability, leukocyte trafficking, and tissue oxygenation. Under selected conditions, these effects may transiently improve immune-cell access to tumor tissue. Conversely, vascular injury and persistent tissue remodeling can worsen hypoxia, fibrosis, and immune exclusion [40,65,85]. CAFs also exhibit complex responses to irradiation. Radiation-induced tissue injury can activate fibroblasts and stimulate ECM remodeling, collagen deposition, and TGF-β signaling. These processes may increase tissue stiffness and establish physical and biochemical barriers to immune-cell migration. Thus, the impact of RT on the stromal compartment may evolve from an acute inflammatory phase toward a chronic tissue-repair program that can contribute to treatment resistance and recurrence [40,65,85]. Macrophages likewise respond dynamically to radiation. Early inflammatory responses may contribute to antigen processing and tissue clearance, whereas persistent injury and repair signals can promote macrophage states associated with angiogenesis, fibrosis, and immunosuppression. The ultimate effect therefore depends on timing and the broader inflammatory context [48,85].

### 6.4. The Abscopal Effect: A Hypothesis-Generating Phenomenon

The abscopal effect refers to regression of tumor lesions outside the irradiated field following localized RT. Although historically recognized as an uncommon phenomenon, experimental studies support the possibility that radiation may stimulate systemic antitumor immunity under appropriate conditions. Local tumor irradiation can increase antigen release and presentation and promote tumor-reactive T-cell responses capable of affecting distant disease [85,90,91,92]. However, spontaneous abscopal responses remain uncommon in routine clinical practice. Combination with ICB has provided a biological strategy for amplifying radiation-associated immune responses, but the magnitude and reproducibility of this effect vary substantially among cancer types, patients, and treatment regimens. Therefore, the abscopal effect should not be presented as an expected clinical consequence of RT or as evidence that adding ICB routinely converts local RT into effective systemic therapy. In breast cancer, it remains an uncommon, hypothesis-generating phenomenon whose frequency, determinants, and clinical relevance are unresolved [91,92,93,94].

### 6.5. Radiotherapy and Immune Checkpoint Blockade: Clinical Evidence and Limitations

RT and ICB have complementary biological mechanisms. Radiation can increase antigen availability and inflammatory signaling, whereas checkpoint inhibitors can prevent inhibitory signaling that limits T-cell activity. This provides a biologically plausible mechanistic rationale for combining the two modalities [84,94]. Preclinical studies have repeatedly demonstrated interactions between RT and checkpoint blockade, and clinical investigations across several tumor types have provided evidence that some patients may benefit from such combinations. However, these observations cannot be assumed to establish efficacy in breast cancer. Breast-cancer-specific clinical data remain limited, heterogeneous, and insufficient to determine whether RT adds benefit to ICB beyond the effects of either modality or accompanying systemic therapy. In breast cancer, the optimal RT dose, fractionation, treated volume, sequence, and timing relative to ICB remain uncertain. Recent reviews and available clinical studies indicate that evidence is currently insufficient to establish a universal optimal sequence or radiation regimen when RT is combined with ICB [16,84,94]. Recent breast-cancer-specific reviews reinforce this point. They identify RT as a potential biological modifier of the breast-cancer immune microenvironment, but the immunological consequences remain dose and context-dependent [95,96]. Accordingly, combinations involving RT and ADCs, PARP inhibitors, STING agonists, TGF-β-directed agents, anti-angiogenic therapy, CDK4/6 inhibitors, or therapeutic vaccines should presently be described as investigational strategies rather than established standards of care [16,17,23,55,87]. Breast-specific trials provide useful but limited evidence. TONIC randomized 67 patients with metastatic TNBC to short induction strategies, including 3 × 8 Gy irradiation, or no induction before nivolumab. Its non-comparative phase II design and the concentration of responses in chemotherapy-induction cohorts do not establish an RT sequencing advantage [97]. Ho and colleagues studied pembrolizumab with RT in 17 patients with metastatic TNBC; responses in unirradiated lesions were observed, but the single-arm design cannot quantify the added benefit of RT [98]. These studies support further investigation, not a standard RT-ICB sequence.

### 6.6. Radiation-Induced Immunosuppression and Research Priorities

Despite its immunostimulatory potential, RT can simultaneously activate mechanisms that suppress durable anti-tumor immunity. Radiation-associated inflammation and tissue damage can promote expression of inhibitory immune pathways, recruitment or expansion of suppressive myeloid and regulatory populations, and production of mediators including TGF-β, VEGF, IL-10, prostaglandins, and adenosine [48,65,85]. Persistent stromal remodeling, ECM deposition, fibrosis, and hypoxia can further restrict lymphocyte trafficking and reinforce an immunosuppressive microenvironment. Radiation can therefore generate a dynamic balance between immune stimulation and compensatory suppression rather than producing a uniformly immunogenic state [40,85]. This duality is central to the development of radiation–immunotherapy combinations. The objective is not simply to enhance radiation-induced inflammation but to preserve beneficial immune activation while limiting the subsequent stromal, metabolic, vascular, and myeloid mechanisms that can drive resistance. The central RT research question is whether a defined dose, fractionation, target volume, and ICB sequence improves clinical outcomes while limiting lymphoid injury and compensatory suppression. Mechanistic and exploratory immune endpoints should be distinguished from efficacy endpoints; Section 10.2 outlines the broader requirements for prospective validation [16,64,85,86,97,98].

## 7. Mechanisms of Immune Escape and Therapeutic Resistance

### 7.1. Immune Escape as a Dynamic Evolutionary Process

Although ICB and rational combination therapies have improved outcomes for selected patients with breast cancer, primary and acquired resistance remain major limitations. Some tumors fail to respond despite evidence of immune infiltration, whereas others initially respond but subsequently progress. These observations indicate that immune resistance rarely reflects a single molecular defect. Instead, it emerges through reciprocal interactions among malignant cells, immune populations, stromal compartments, vasculature, metabolism, and treatment-induced selective pressures [4,79,99,100]. Immune surveillance itself can shape tumor evolution by selecting malignant subclones with reduced immunogenicity or enhanced resistance to immune-mediated elimination. During treatment, tumor cells may further alter antigen presentation, inflammatory signaling, checkpoint-ligand expression, metabolic programs, and interactions with surrounding stromal and immune populations. Thus, immune escape is best understood as an evolving process in which tumor-intrinsic adaptations and microenvironmental remodeling cooperate to limit effective antitumor immunity [79,99,101]. This system-level model has important therapeutic implications (Figure 6). Resistance may arise at multiple stages of the cancer–immunity cycle, including antigen generation and presentation, T-cell priming, lymphocyte trafficking, tumor infiltration, malignant-cell recognition, effector function, and establishment of immune memory. Consequently, successful therapeutic strategies may need to identify the dominant resistance mechanisms operating within an individual tumor rather than assuming that failure of PD-1/PD-L1 blockade reflects inadequate checkpoint inhibition alone [4,79,99,100].

### 7.2. Tumor-Intrinsic Mechanisms of Immune Resistance

Tumor cells can evade immune destruction through multiple intrinsic mechanisms. Impaired antigen presentation, including reduced major histocompatibility complex class I (MHC-I) expression, β2-microglobulin loss, defective antigen-processing machinery, altered interferon signaling, and changes in peptide presentation, can diminish recognition by cytotoxic CD8^+^ T cells despite continued expression of TAAs [101,102,103]. However, much of the mechanistic and clinical evidence for these alterations as causes of immunotherapy resistance derives from melanoma, lung cancer, other tumor types, and preclinical models. Their prevalence and clinical significance in breast cancer remain incompletely established. Genomic and epigenetic evolution may further reshape the antigenic landscape. Immune selection can eliminate highly immunogenic clones while favoring subclones with fewer recognizable neo-antigens or impaired antigen presentation, a process broadly described as cancer immunoediting. Such changes may occur during natural tumor evolution and can be accelerated by therapeutic pressure [101,104]. Nevertheless, direct longitudinal evidence linking immunoediting to acquired checkpoint-inhibitor resistance in breast cancer remains limited.

Oncogenic signaling also influences immune responsiveness. Aberrant WNT/β-catenin, PI3K-AKT-mTOR (including alterations associated with PTEN loss), MYC, RAS-MAPK, STAT3, NF-κB, and TGF-β signaling can affect cytokine and chemokine production, antigen presentation, dendritic-cell recruitment, and immune-checkpoint expression [105,106,107,108]. These mechanisms have been defined most convincingly in melanoma, lung cancer, and mouse models. Breast cancer studies support functional links between oncogenic signaling and immune regulation, but clinical validation of individual pathways as determinants of immunotherapy resistance remains variable and, for several pathways, preliminary. Breast cancer-associated signaling programs add another layer of complexity. Estrogen-receptor and HER2 signaling, DNA-damage responses, and cell-cycle regulation can influence interferon pathways, inflammatory signaling, antigen presentation, and tumor–immune interactions. These observations provide a rationale for combining immunotherapy with endocrine therapy, HER2-directed treatment, DNA-damage response inhibitors, or CDK4/6 inhibitors in selected settings [14,17,26]. However, for many such combinations, mechanistic evidence exceeds current evidence of clinical benefit, and efficacy may differ across breast cancer subtypes. Tumor and microenvironmental cells can additionally engage inhibitory pathways beyond PD-1/PD-L1, including TIGIT, TIM-3, VISTA, CD47–SIRPα, B7-H3, and B7-H4. These pathways may contribute to dysfunctional or exhausted immune states and acquired resistance [77,100,109,110]. Yet detailed T-cell exhaustion hierarchies and many mechanisms of metabolic or acquired checkpoint resistance have been characterized principally in non-breast tumors or experimental models. VISTA is of particular interest because of its immunoregulatory functions in both T-cell and myeloid compartments, although its clinical relevance and therapeutic role in breast cancer remain investigational. Overall, these mechanisms should be regarded as biologically plausible and potentially relevant to breast cancer rather than as uniformly demonstrated clinical resistance mechanisms in this disease.

### 7.3. T-Cell Dysfunction and Progressive Exhaustion

Functional impairment of tumor-reactive T lymphocytes is a major feature of chronic antitumor immune responses. Persistent antigen exposure, inflammatory signaling, metabolic stress, and suppressive microenvironmental factors can drive T cells toward dysfunctional states characterized by reduced proliferative capacity, impaired cytokine production, altered metabolism, and expression of inhibitory receptors including PD-1, LAG-3, TIM-3, TIGIT, CTLA-4, and other regulatory molecules [43,111,112]. The progenitor and terminally exhausted states described in Section 3.4 have different capacities to respond to checkpoint blockade [42,112,113]. Single-cell analysis of breast cancers treated with anti-PD-1 therapy similarly demonstrates heterogeneous T-cell states and treatment-associated remodeling of immune populations. These observations support the relevance of T-cell differentiation state in breast cancer while also emphasizing that mechanisms initially established in other tumor models should not automatically be assumed to operate identically across all breast cancer subtypes [8]. Preservation of tumor-reactive progenitor populations may therefore be important for durable responses. Future therapeutic strategies may need not only to reverse inhibitory signaling but also to maintain or restore the cellular populations capable of generating sustained effector responses [42,43,113].

### 7.4. Myeloid Cell Plasticity and Immunosuppressive Niches

Macrophage programs associated with tumor progression can promote angiogenesis, tissue remodeling, immune suppression, and metastatic dissemination through IL-10, TGF-β, VEGF, arginase-associated metabolism, prostaglandins, matrix-remodeling enzymes, and other mediators. These functions can suppress T-cell activity directly while simultaneously modifying vascular and stromal compartments [48,49] (Figure 7). MDSCs can further inhibit adaptive immunity through arginine depletion, reactive oxygen and nitrogen species, suppressive cytokines, and interactions with Tregs, DCs, and NK cells [50]. Spatial organization is also important. Myeloid-rich regions can coexist with abundant lymphocyte infiltration while limiting productive tumor-T-cell interactions. Thus, myeloid suppression should be considered both a functional and spatial mechanism of resistance. Therapeutic approaches targeting macrophage recruitment, polarization, and phagocytic checkpoints, including the CD47-SIRPα axis, remain active areas of investigation [6,48,49,110].

### 7.5. Stromal Remodeling and Immune Exclusion

The stromal compartment actively regulates antitumor immunity. CAFs influence ECM deposition, collagen organization, tissue stiffness, cytokine and chemokine gradients, vascular architecture, and immune-cell trafficking. Importantly, CAFs are heterogeneous, and individual fibroblast states can exert different or even opposing biological functions [12,52,53]. Among the signaling networks connecting stromal and immune compartments, CXCL12-CXCR4 signaling can contribute to stromal and myeloid mechanisms of immune exclusion, providing a rationale for therapeutic CXCR4 inhibition to improve antitumor immune responses [51]. Fibroblast activation protein (FAP), expressed by selected activated fibroblast populations, has also emerged as a potential therapeutic target for modifying tumor-promoting stromal compartments. FAP-directed strategies, including antibody-, radioligand-, vaccine-, and cellular therapy-based approaches, are under investigation, although their clinical role in breast cancer remains to be established [12,52,53,54]. TGF-β represents an important link between stromal activation and immune exclusion. Experimental and clinical studies across tumor types have demonstrated that TGF-β associated stromal programs can restrict T-cell access to malignant epithelial compartments and reduce responsiveness to checkpoint blockade [80]. Breast-specific spatial evidence is discussed in Section 3.5. Fibrotic remodeling can additionally increase interstitial pressure, impair vascular function and drug delivery, and worsen tissue hypoxia. Stromal resistance should therefore be considered an integrated biomechanical and immunological process rather than simply a physical barrier to T-cell migration [12,40,52].

### 7.6. Metabolic Competition Within the TME

Metabolic conditions within tumors profoundly influence immune-cell function. Rapidly proliferating malignant cells and activated stromal populations compete with immune cells for glucose, amino acids, lipids, and oxygen. Increased glycolysis can generate lactate and extracellular acidification, while nutrient depletion can impair T-cell proliferation, cytokine production, and cytotoxic activity [57,58,59]. Hypoxia further modifies the immune ecosystem through HIF-dependent signaling, VEGF production, altered myeloid-cell function, and accumulation of immunosuppressive metabolites such as adenosine. Additional pathways involving tryptophan metabolism, arginine availability, lipid metabolism, cholesterol homeostasis, mitochondrial function, and oxidative phosphorylation can influence both innate and adaptive immunity [57,58,59,60]. These findings demonstrate that immune resistance is not solely a consequence of checkpoint signaling. Even appropriately activated tumor-reactive lymphocytes may fail to function effectively when they enter a metabolically hostile microenvironment [57,58,59,60]. The therapeutic implications are substantial, but many immunometabolic interventions remain investigational. For example, clinical experience with indoleamine 2,3-dioxygenase 1 (IDO1) inhibition has illustrated that compelling biological rationale does not necessarily translate into successful combination therapy. The negative ECHO-301/KEYNOTE-252 trial was conducted in melanoma; it illustrates a translational limitation rather than a direct breast-cancer efficacy result [114].

### 7.7. Chronic Inflammation and Compensatory Immune Suppression

Inflammation can exert opposing effects during cancer therapy. Acute inflammatory responses induced by tumor-cell death may enhance antigen presentation and immune activation, whereas persistent inflammation can promote chronic tissue repair, fibroblast activation, angiogenesis, myeloid-cell recruitment, and immunosuppressive cytokine production [13,23,48]. TGF-β is a central mediator linking tissue repair, fibrosis, and immune suppression. Persistent TGF-β signaling can promote ECM deposition, immune exclusion, epithelial–mesenchymal plasticity, and suppression of cytotoxic lymphocyte function [23,63,80]. VEGF, colony stimulating factor 1 (CSF1)-associated signaling, prostaglandins, IL-10, and adenosine can contribute additional overlapping suppressive networks. Rather than acting independently, these pathways frequently reinforce one another, helping to explain why inhibition of a single suppressive mechanism may produce incomplete or transient therapeutic benefit [23,48,60].

## 8. Emerging Therapeutic Strategies and Rational Combination Approaches

### 8.1. Moving Beyond Single-Agent Immunotherapy

The limited efficacy of ICB monotherapy across substantial portions of the breast cancer population has highlighted the complexity of tumor immune regulation. Clinical and translational evidence indicates that resistance can arise through coordinated interactions among malignant cells, stromal compartments, innate and adaptive immune populations, vascular biology, and metabolism rather than through dysfunction of an individual immune checkpoint alone. Consequently, therapeutic development is increasingly shifting toward rational combinations designed to address multiple, complementary barriers to effective antitumor immunity [1,26,79]. This transition reflects a broader conceptual change in breast cancer immunotherapy. Rather than asking whether a tumor is intrinsically sensitive or resistant to checkpoint blockade, current approaches increasingly seek to identify the dominant biological barriers within an individual tumor and determine which can be therapeutically reversed. The objective of combination therapy is therefore not simply to increase immune activation, but to remodel immune-excluded or immunosuppressed tumors toward states that permit sustained antigen presentation, lymphocyte trafficking, tumor-cell recognition, and effector function [1,26,79] (Table 4).

### 8.2. Combining Immunotherapy with Chemotherapy

Chemotherapy was historically viewed primarily as a cytotoxic strategy for reducing tumor burden. Increasing evidence demonstrates, however, that selected cytotoxic agents can also modify antitumor immunity. Selected agents induce the immunogenic cell-death pathways discussed in Section 6.2 [15,63,87]. Chemotherapy can also alter suppressive immune-cell populations and inflammatory signaling, creating a transient TME that may be more responsive to ICB. These effects provide a biological rationale for combining chemotherapy with ICB, particularly in TNBC, where randomized trials have demonstrated clinically meaningful benefits in both metastatic and early-stage disease [10,11,18,31]. However, clinical evidence indicates that chemotherapy regimens are not interchangeable as immune-priming partners. The immunologic effects, efficacy, and toxicity of these combinations may vary according to the cytotoxic agent, dose, formulation, and treatment schedule, including the choice of taxane and the use of platinum-based therapy. The discordant results of IMpassion130 and IMpassion131 illustrate that a strong biological rationale does not necessarily translate into clinical benefit across different treatment platforms [27,29,30]. Future studies should therefore identify the cytotoxic agents, doses, and treatment sequences that produce the most favorable immunologic effects while minimizing lymphodepletion, tissue injury, and compensatory immunosuppression [15,17,63,87].

### 8.3. Radiotherapy–Immunotherapy Combinations

Because RT can both stimulate and suppress antitumor immunity, RT-ICB combinations require context-specific optimization of dose, fractionation, sequence, and patient selection; the mechanistic and clinical evidence is detailed in Section 6.

### 8.4. Endocrine Therapy and Immune Modulation

The interaction between endocrine signaling and tumor immunity is increasingly relevant in HR^+^/HER2^−^ breast cancer. Estrogen receptor signaling can influence inflammatory pathways, cytokine production, macrophage states, antigen presentation, and lymphocyte recruitment. Endocrine therapy may therefore modify the immune ecosystem in addition to suppressing tumor-cell proliferation [115]. These observations provide a rationale for combining endocrine therapy with immunotherapy in selected patients. The recent KEYNOTE-756 trial demonstrated that adding pembrolizumab to neoadjuvant chemotherapy increased pCR in patients with high-risk, early-stage ER^+^/HER2^-^ breast cancer. Importantly, these data support evaluation of perioperative immunotherapy in high-risk HR^+^ disease, rather than establishing checkpoint blockade as a routine treatment for unselected HR^+^/HER2^-^ breast cancer [36]. Future studies integrating endocrine treatment with longitudinal immune profiling and spatial analysis may help determine which immune states are most amenable to therapeutic modulation.

### 8.5. CDK4/6 Inhibitors as Immunomodulatory Agents

CDK4/6 inhibitors have transformed the treatment of HR^+^/HER2^−^ breast cancer and have generated substantial interest as immunomodulatory agents. Preclinical studies demonstrate that CDK4/6 inhibition can enhance antigen presentation, increase interferon-associated signaling, alter regulatory T-cell proliferation, and promote antitumor immune activity [14]. These findings provide a compelling biological rationale for combining CDK4/6 inhibition with checkpoint blockade. Nevertheless, translation from mechanistic studies into broadly effective clinical combinations has been more challenging than initially anticipated. For example, a breast-cancer phase Ib study of abemaciclib plus pembrolizumab reported substantial hepatic and pulmonary toxicity, and its benefit–risk findings did not support further development of that combination [116]. An additional consideration is that prolonged CDK4/6 inhibition can impose selective pressures that alter tumor-cell state, stromal signaling, and cell-cycle biology. Future studies should therefore focus not only on concurrent administration but also on identifying treatment sequences that exploit periods of enhanced immunogenicity while limiting adaptive resistance.

### 8.6. DNA Damage Response Inhibitors

Defects in DNA repair provide an important biological link between tumor genomics and immune activation. PARP inhibition exploits DNA-repair vulnerabilities, particularly in BRCA1/2-mutated breast cancer. The established olaparib trials demonstrate PARP-inhibitor efficacy, not the added value of ICB [17,117,118]. Homologous recombination deficiency (HRD) provides a biological context for investigating combinations, but should not be treated as an independently validated selector for ICB. However, the clinical benefit of PARP-immunotherapy combinations has not been uniformly demonstrated across breast cancer populations. PARP inhibitors themselves have established clinical value in genetically defined subsets, particularly BRCA1/2-mutated disease, but their addition to immunotherapy remains an area of active investigation. Additional DDR targets, including ATR, ATM, WEE1, CHK1, and DNA-PK, may influence tumor immunogenicity through effects on DNA damage, replication stress, and innate immune sensing. At present, most such combinations remain investigational and require prospective clinical validation [17,119].

### 8.7. Antibody–Drug Conjugates and Immune Remodeling

ADCs have become major therapeutic platforms across HER2^+^, HER2-low, HER2-ultralow, and TNBC populations. Trials of trastuzumab deruxtecan and sacituzumab govitecan establish ADC efficacy through targeted cytotoxic delivery, including the long-term DESTINY-Breast04 findings [69,70,120,121]. Potential effects on antigen availability and immune remodeling remain distinct mechanistic questions. The immunological consequences of ADC therapy vary according to target antigen, linker properties, payload, intracellular trafficking, and bystander activity. It is therefore premature to assume that all ADCs function equivalently as immune-priming agents [75,122]. Clinical development is increasingly exploring combinations of ADCs with immune checkpoint blockade. The phase Ib/II BEGONIA study demonstrated encouraging activity with the TROP2-directed ADC datopotamab deruxtecan (Dato-DXd) plus durvalumab as first-line therapy for unresectable locally advanced or metastatic TNBC, providing clinical rationale for further evaluation of this combination [122]. The phase III TROPION-Breast05 trial is evaluating Dato-DXd with or without durvalumab versus chemotherapy plus pembrolizumab in patients with PD-L1-high locally recurrent inoperable or metastatic TNBC [123]. These studies illustrate the growing interest in integrating ADC-mediated tumor-cell killing with checkpoint blockade, although definitive clinical benefit cannot be assumed across ADCs, immune checkpoint inhibitors, or disease settings. More broadly, the therapeutic landscape of ADCs continues to expand independently of immunotherapy combinations. In DESTINY-Breast06 [124], trastuzumab deruxtecan (T-DXd) significantly improved progression-free survival compared with physician’s-choice chemotherapy in the HER2-low primary analysis population; benefit was also observed in the exploratory HER2-ultralow cohort [124]. More recently, DESTINY-Breast11 demonstrated that neoadjuvant T-DXd followed by paclitaxel, trastuzumab, and pertuzumab (THP) significantly increased pCR compared with dose-dense anthracycline-based chemotherapy followed by THP in patients with high-risk HER2-positive early breast cancer [125]. These trials establish the expanding clinical reach of ADCs but should be distinguished from trials specifically evaluating ADC-checkpoint inhibitor combinations. These developments strengthen the rationale for investigating how ADC-mediated tumor-cell killing and microenvironmental remodeling might be exploited therapeutically. However, predictive biomarkers capable of identifying patients most likely to benefit specifically from ADC-checkpoint combinations remain an important unmet need. ASCENT-04/KEYNOTE-D19 provides randomized evidence for a specific ADC-ICB regimen; its results and June 2026 FDA indication are summarized in Table 3.

### 8.8. Targeting Stromal and Myeloid Compartments

Recognition of CAF and myeloid plasticity as important mechanisms of immune exclusion has expanded therapeutic development beyond tumor cells and lymphocytes. Investigational strategies include modulation of TGF-β, CSF1R, CXCR4-CXCL12, focal adhesion kinase, FAP, ECM remodeling, and related stromal pathways [12,48,52,80]. Myeloid-directed approaches include strategies targeting CSF1/CSF1R signaling, CD47-SIRPα interactions, macrophage recruitment or polarization, and MDSC-associated pathways. The objective is to restore antigen presentation, reduce suppressive signaling, and facilitate productive T-cell-tumor interactions [48,49,50]. Importantly, these strategies must account for the functional heterogeneity of CAFs and macrophages. Broad elimination of stromal or myeloid populations may have unintended consequences because some populations also support antigen presentation, tissue integrity, and immune activation. More selective reprogramming may therefore be preferable to indiscriminate depletion [12,48,49,52].

### 8.9. Activating Innate Immunity

Increasing attention has focused on pharmacological activation of innate immune sensing. Agonists of STING, Toll-like receptors, RIG-I-like receptors, and related pattern-recognition pathways can promote dendritic-cell activation, type I interferon production, NK-cell activity, and subsequent T-cell priming [46,64,88]. Personalized neoantigen vaccines and oncolytic viruses represent complementary strategies that can increase tumor immunogenicity and generate or expand tumor-reactive T-cell populations [4,126,127]. These approaches are particularly attractive for tumors with inadequate endogenous antigen presentation or limited baseline T-cell priming. However, systemic innate immune activation can produce toxicity and may trigger compensatory immunosuppressive pathways. The most effective strategies will therefore likely require tumor-selective delivery, biomarker-based patient selection, or combination with checkpoint blockade to sustain adaptive immune responses after initial innate activation [46,64,88,127].

### 8.10. Targeting Immunometabolism

Metabolic remodeling is another potential therapeutic axis. Tumor-associated hypoxia, glucose depletion, lactate accumulation, adenosine signaling, altered amino-acid availability, lipid metabolism, and mitochondrial dysfunction can impair immune-cell function within the TME [57,58,59,60]. Investigational approaches have targeted IDO1, adenosine receptors, CD39/CD73, HIF signaling, oxidative metabolism, lipid utilization, and related pathways [57,58,59,60,114]. Importantly, the failure of IDO1 inhibition to improve outcomes when combined with PD-1 blockade in a phase III melanoma trial illustrates that compelling metabolic rationale does not necessarily translate into clinical benefit and reinforces the need for biomarker-guided development of immunometabolic strategies [114]. The underlying objective is to restore metabolic conditions that permit sustained immune-cell activation rather than merely increasing the number of immune cells entering the tumor [57,58,59,60]. The clinical translation of immunometabolic therapies remains challenging. Several biologically promising strategies have not produced the expected clinical benefit, emphasizing the need to identify the specific metabolic dependencies operating within individual tumor ecosystems rather than applying broad metabolic inhibition indiscriminately [57,58,59,60].

### 8.11. Precision Combination Immunotherapy

The marked heterogeneity of breast cancer makes a universal combination strategy unlikely to be effective. Instead, precision immunotherapy will increasingly depend on multidimensional characterization of tumor biology before and during treatment [6,7,8,21,53]. Single-cell sequencing, spatial transcriptomics, multiplex imaging, proteomics, ctDNA, digital pathology, radiomics, and AI-based computational methods can provide complementary information on malignant-cell states, immune populations, stromal organization, vascular biology, and treatment-induced evolution [6,7,8,53]. The major opportunity is to integrate these modalities into actionable models that identify the dominant resistance mechanism within an individual tumor. For example, a tumor characterized primarily by stromal exclusion may require a different combination from one dominated by antigen-presentation defects, myeloid suppression, or metabolic dysfunction. Such an approach would move therapeutic selection beyond static subtype classification toward functional characterization of tumor ecosystem state. Rational combinations should address complementary, biologically demonstrated resistance mechanisms rather than simply add agents. The mechanistic rationale and current clinical status of representative combinations are summarized in Table 5.

## 9. Biomarkers for Precision Immunotherapy

### 9.1. From Static Biomarkers to Dynamic Immune Profiling

The expanding role of immunotherapy in breast cancer has intensified efforts to identify biomarkers that can predict therapeutic benefit, refine patient selection, monitor treatment response, and detect emerging resistance. Although ICB produces clinically meaningful benefit in selected populations, substantial heterogeneity persists both within and across breast cancer subtypes. Consequently, no single biomarker adequately captures the complex biological processes that determine immune responsiveness [4,9]. Initial biomarker development focused primarily on PD-L1 expression, TMB, and TILs. These measures remain clinically informative, particularly PD-L1 in selected metastatic TNBC settings and TILs as a prognostic and treatment-response marker in several breast cancer subtypes. Nevertheless, their predictive performance varies according to disease stage, assay methodology, treatment regimen, and biological context [4,5,18,130] (Table 6). Increasing evidence indicates that immune responsiveness emerges from interactions among malignant cells, stromal compartments, innate and adaptive immunity, metabolism, vasculature, and treatment-induced remodeling. Biomarker development is therefore shifting from isolated baseline measurements toward multidimensional assessment of the evolving tumor ecosystem [4].

A prognostic biomarker identifies differences in outcome; a predictive biomarker identifies differences in the relative benefit of a particular treatment. Association with response in a treated cohort is insufficient to establish treatment-specific prediction. That claim requires appropriate comparative evidence, ideally a prespecified treatment-by-biomarker interaction with independent validation. Clinical utility additionally requires evidence that acting on a biomarker improves decisions or outcomes [4,9,131].

**Table 6:** Biomarkers for Precision Immunotherapy in Breast Cancer.

Biomarker	Biological Information	Clinical Utility	Major Limitations	References
PD-L1 expression	Reflects checkpoint-ligand expression and inflammatory signaling	Actionable for specific CPS ≥10 advanced-TNBC regimens; not required for perioperative pembrolizumab in eligible early TNBC.	Dynamic expression, assay/platform differences, spatial heterogeneity, incomplete sensitivity and specificity	[4,10,11,18,75]
Tumor-infiltrating lymphocytes (TILs)	Measures endogenous immune infiltration	Prognostic and associated with response; not independently validated to select incremental ICB benefit.	Does not adequately describe spatial localization, cellular composition, or functional state; not a universal standalone ICB biomarker	[5,130,132]
Tumor mutational burden (TMB)	Approximate genomic mutation load and potential neoantigen generation	Predictive in selected tumor contexts	Generally modest in breast cancer; mutation number does not capture neoantigen quality, clonality, HLA presentation, or immune editing	[4,133,134]
BRCA1/2 mutation/HRD	Defective homologous recombination and DNA repair	Established biomarker for PARP-inhibitor therapy; provides rationale for selected immunotherapy combinations	Applies to molecularly defined subsets; immunotherapy-predictive value is not established solely by BRCA status	[17,117,118]
Interferon-γ/T-cell-inflamed gene signatures	Reflect inflammatory and immune-effector programs	Exploratory response associations; breast-specific treatment-predictive utility not established.	Dynamic and treatment-dependent; assay and threshold standardization remain challenges	[135,136,137]
Tertiary lymphoid structures (TLSs)	Organized local immune activation and B/T-cell interactions	Prognostic or response associations in research cohorts; not a validated routine ICB-selection test in breast cancer.	Assessment and maturation criteria are not yet standardized for routine clinical use	[19,20,45]
ctDNA	Molecular measurement of residual tumor burden and tumor evolution	Longitudinal response and recurrence-risk assessment; increasingly important in early breast cancer	Assay sensitivity, timing, low shedding, and thresholds affect interpretation; both tumor-informed and tumor-agnostic methods exist. ctDNA-guided ICB escalation, de-escalation, and sequencing remain investigational.	[138,139,140,141]
Spatial transcriptomics/multiplex imaging	Cellular localization and tissue architecture	Research biomarker for immune neighborhoods, exclusion, TLSs, and tumor–stromal interactions	Cost, tissue requirements, analytical complexity, limited standardization	[7,8,21]
Single-cell multiomics	Cellular heterogeneity and functional states	Defines immune, tumor, stromal, and treatment-induced states; particularly valuable for mechanistic biomarker development	Computationally intensive; sampling and tissue requirements; limited routine clinical validation	[6,7,8]
AI/digital pathology models	Integration of morphology, spatial patterns and molecular information	Investigational precision stratification and outcome prediction	Requires external validation, standardized datasets, interpretability, and demonstration of clinical utility	[131,142,143,144,145]

### 9.2. PD-L1 Expression: An Imperfect but Clinically Relevant Biomarker

PD-L1 remains the most clinically established biomarker for selecting patients with metastatic TNBC for specific checkpoint-inhibitor regimens. In KEYNOTE-355, pembrolizumab plus chemotherapy significantly improved OS compared with chemotherapy in patients whose tumors expressed PD-L1 with a CPS of at least 10. In contrast, the CPS ≥1 population did not demonstrate a statistically significant overall-survival benefit in the final analysis [10]. These findings illustrate both the clinical utility and limitations of PD-L1. Responses can occur in some tumors with low or absent PD-L1 expression, whereas many PD-L1^+^ tumors fail to respond. PD-L1 expression is also dynamic and may be influenced by inflammatory cytokines, interferon signaling, chemotherapy, radiotherapy, and other therapeutic interventions. Intratumoral heterogeneity, differences between primary and metastatic lesions, temporal variation, and assay-specific scoring systems further complicate interpretation [4,10,131]. Accordingly, PD-L1 should be regarded as a context-dependent predictive biomarker rather than a universal measure of immune competence. In contrast, PD-L1 positivity is not a prerequisite for perioperative pembrolizumab in eligible high-risk early TNBC [11,18]. Assay, scoring system, cutoff, regimen, and disease setting must therefore accompany any claim of PD-L1 utility.

### 9.3. Tumor-Infiltrating Lymphocytes

TILs provide an important measure of endogenous anti-tumor immunity. Higher TIL density is associated with favorable prognosis and treatment response, particularly in TNBC and HER2+ breast cancer. In the neoadjuvant setting, increased TILs are also associated with a higher likelihood of pCR to systemic therapy [5,130,132]. However, TIL abundance alone provides limited information about immune functionality. Lymphocytes may be concentrated within stromal compartments rather than in direct contact with malignant cells, while exhausted, regulatory, or otherwise dysfunctional populations may coexist with apparently abundant immune infiltration. Spatial organization, cellular composition, differentiation state, and functional activity therefore provide important complementary information [6,7,8]. Future TIL-based biomarkers will likely move beyond percentage measurements toward characterization of specific immune populations, spatial relationships, clonality, and functional state. These prognostic and response associations do not by themselves validate TILs as a standalone selector for the incremental benefit of ICB [5,130].

### 9.4. Tumor Mutational Burden and Neoantigen Landscape

TMB has demonstrated predictive value across multiple malignancies because increased mutational burden can increase the probability of generating immunogenic neoantigens. However, its predictive value is context-dependent and less consistently informative in breast cancer than in tumor types characterized by very high mutational burden [104,133]. Most breast cancers have relatively modest TMB, although subsets of TNBC and tumors with DNA-repair abnormalities may display increased genomic instability. Importantly, mutation number alone does not determine immunogenicity. Neoantigen quality and clonality, human leukocyte antigen (HLA) genotype, antigen-processing capacity, immune editing, and the ability of tumor-reactive T cells to access malignant cells all influence effective immune recognition [104,134,135]. Consequently, future genomic biomarkers may need to move beyond aggregate TMB toward integrated characterization of neoantigen quality, clonality, antigen presentation, and immune contexture.

### 9.5. Gene-Expression Signatures

Transcriptomic biomarkers provide a broader representation of tumor immune states than single molecular markers. Interferon-γ associated signatures, T-cell-inflamed gene-expression profiles, cytolytic activity measures, chemokine programs, and antigen-presentation pathways have each been associated with response to checkpoint blockade in different cancer settings [131,136,137].

Conversely, transcriptional programs associated with TGF-β signaling, angiogenesis, epithelial–mesenchymal plasticity, stromal remodeling, and myeloid inflammation may characterize immune-excluded or treatment-resistant states [80,105]. These findings support development of composite signatures that simultaneously assess immune activation and immune suppression rather than interpreting a single pathway in isolation. However, many transcriptomic signatures remain investigational and require prospective validation before routine clinical implementation [80,105,131,136,137]. In breast cancer, IFN-γ signatures remain candidate treatment-response markers rather than established tests for choosing ICB over an otherwise comparable regimen.

### 9.6. Single-Cell Technologies Reveal Immune Heterogeneity

Single-cell RNA sequencing and related single-cell multiomic technologies have transformed the characterization of breast cancer immune ecosystems. These approaches reveal heterogeneity among malignant cells, macrophages, dendritic cells, NK cells, fibroblasts, endothelial cells, and lymphocyte populations that cannot be resolved using bulk sequencing [6,8,21]. In particular, single-cell studies have identified distinct populations of progenitor-exhausted, terminally exhausted, proliferating, tissue-resident memory, regulatory, and other T-cell states. The biological significance of these populations differs according to disease context and treatment, but their identification provides an important framework for understanding response and resistance [8,42,113]. Single-cell profiling of paired pretreatment and on-treatment biopsies can characterize treatment-associated immune remodeling. In breast cancer, Bassez et al. identified changes in T-cell states and clonal expansion following anti-PD-1 treatment, demonstrating the value of this approach for studying early immune responses [6,7,8,21]. Whether serial profiling can distinguish transient activation from durable immune reprogramming or identify resistance before clinical progression requires prospective validation. At present, however, the clinical application of single-cell biomarkers remains primarily investigational because standardized assays, thresholds, tissue requirements, and prospective clinical validation are still evolving [6,7,8,21].

### 9.7. Spatial Biomarkers and Tumor Architecture

Spatial biology has emerged as an important complement to conventional histopathology and bulk molecular profiling. Spatial transcriptomics, multiplex immunofluorescence, imaging mass cytometry, digital spatial profiling, and highly multiplexed proteomic imaging allow cellular identity to be evaluated together with tissue organization [21,53]. These approaches demonstrate that immune function depends not only on the presence of specific cell populations but also on their spatial relationships. Interactions among dendritic cells, cytotoxic T cells, B cells, macrophages, fibroblasts, endothelial cells, and malignant cells may influence whether immune activation translates into effective tumor-cell killing. Similarly, CAF-rich barriers, abnormal vasculature, ECM architecture, and organized TLSs can alter immune-cell trafficking and local immune function [7,21,22,45,53]. Although spatial biomarkers may provide information beyond conventional histopathological measurements in selected research settings, prospective clinical validation, assay standardization, and demonstration of reproducibility will be required before their routine clinical implementation [7,21,22,53]. TLSs and spatial immune neighborhoods should likewise be described as exploratory or prognostic associations unless treatment-specific predictive value has been demonstrated.

### 9.8. Liquid Biopsies and Longitudinal Monitoring

Liquid biopsy approaches offer an important opportunity to monitor tumor evolution without repeated tissue sampling. ctDNA is increasingly being investigated as a marker of molecular response, residual disease, treatment resistance, and recurrence risk in early breast cancer [138,139,140,141]. Recent 2025 studies provide particularly strong support for the longitudinal application of ctDNA. A tumor-informed study of patients receiving neoadjuvant therapy demonstrated that persistent mid-treatment ctDNA detection was associated with recurrence, while postoperative detection showed a very high positive predictive value for recurrence in the analyzed cohort [139]. A separate 2025 analysis demonstrated that ctDNA dynamics could improve risk stratification among patients with residual disease after neoadjuvant therapy and that early ctDNA clearance was associated with favorable response, including in patients receiving immunotherapy [140]. These findings support a shift from static measurement toward longitudinal molecular monitoring. Nevertheless, ctDNA-guided treatment modification remains an evolving clinical strategy, and prospective interventional studies are required before routine treatment escalation or de-escalation based solely on ctDNA can be broadly recommended [138,139,140,141]. The earlier metastatic-breast-cancer ctDNA study [146] addresses a different clinical setting and should not be taken as evidence for ctDNA-guided immunotherapy decisions in early disease.

### 9.9. Artificial Intelligence and Integrated Biomarker Platforms

Artificial intelligence (AI) and machine learning provide increasingly powerful approaches for integrating multiple biomarker modalities. Digital pathology, radiomics, genomics, transcriptomics, proteomics, spatial profiling, and clinical variables can be combined to identify complex patterns associated with treatment response [142,143,144,145]. Rather than replacing individual biomarkers, AI-based models may integrate complementary information and identify relationships among tumor morphology, immune composition, spatial organization, and treatment-induced changes that are difficult to detect using conventional analyses [142,143,144,145]. The major challenge for AI is validation.

AI models may be sensitive to differences in patient populations, imaging platforms, tissue preparation, sequencing technologies, and clinical workflows. Prospective multicenter validation, standardized data pipelines, interpretability, and rigorous assessment of generalizability will therefore be essential before widespread clinical implementation [142,143,144,145]. High discrimination in a retrospective dataset does not establish that model-guided escalation, de-escalation, or sequencing improves patient outcomes. Such decisions require prospectively tested algorithms and clinical utility studies.

### 9.10. Toward Precision Immunotherapy Guided by Systems Biology

Future precision immunotherapy will require integrated characterization of the tumor ecosystem rather than reliance on isolated biomarkers. Tumor genomics, antigenicity, immune composition, stromal architecture, spatial organization, vascular biology, metabolic state, circulating biomarkers, and treatment-induced temporal changes should be considered complementary dimensions of a unified biological framework [7,21,45,131,147] (Figure 8). The appropriate role of individual biomarkers will also depend on clinical context. PD-L1 currently provides actionable information for selected metastatic TNBC treatment decisions; TILs have prognostic and treatment-response associations, without established standalone utility for selecting ICB; ctDNA is emerging as a promising longitudinal biomarker; and single-cell and spatial approaches are increasingly valuable for mechanistic discovery. No single platform should yet be considered sufficient to define precision immunotherapy across all breast cancer subtypes [5,7,9,10,130,138,141]. The ultimate objective is therefore a dynamic, multimodal biomarker strategy that can identify the biological state of an individual tumor, determine its dominant mechanisms of immune sensitivity or resistance, and monitor those mechanisms during treatment. Such an approach could improve patient selection, optimize therapeutic sequencing, identify resistance earlier, and support adaptive treatment strategies designed around the evolving biology of each tumor.

## 10. Future Perspectives and Conclusions

The evidence reviewed above supports immune responsiveness as a dynamic biological property and TME plasticity as a source of therapeutic hypotheses. Established clinical benefit remains regimen- and population-specific; the independent clinical contribution of treatment timing requires direct testing.

### 10.1. The Future of Precision Immunotherapy

The extraordinary biological heterogeneity of breast cancer remains one of the greatest challenges to precision immunotherapy. Substantial heterogeneity exists not only between patients but also among distinct regions and cellular populations within individual tumors [6,7,8,21]. Current biomarkers, including PD-L1 expression, TMB, and TILs, provide important biological and clinical information but incompletely capture the complexity of immune regulation. Future precision immunotherapy may benefit from multidimensional characterization that integrates tumor genomics and epigenetics with transcriptomics, proteomics, metabolomics, immune phenotyping, spatial organization, vascular biology, ECM remodeling, and treatment-induced temporal changes [7,9,21,131,147]. Single-cell sequencing, spatial transcriptomics, multiplex imaging, liquid biopsy, ctDNA, radiomics, digital pathology, and AI-based computational approaches now provide unprecedented opportunities to characterize tumor ecosystems at cellular and spatial resolution. Their greatest value may lie not in replacing conventional biomarkers but in integrating complementary dimensions of tumor biology into dynamic models of treatment response and resistance [21,45,138,139,140,142,143,144,145].

### 10.2. Challenges for Clinical Translation

Despite remarkable progress, several challenges must be addressed before precision immunotherapy can be broadly implemented. First, standardized methodologies are needed to integrate multiomic, spatial, imaging, and longitudinal datasets into clinically actionable biomarkers. Differences in tissue acquisition, assay platforms, computational pipelines, scoring systems, and analytical thresholds currently limit direct comparison among studies [7,21,53,131,147]. Second, prospective clinical trials should increasingly incorporate longitudinal biological sampling to characterize how immune and stromal states evolve during treatment. Serial tissue sampling may provide particularly valuable information, although feasibility, patient burden, tissue accessibility, and sampling bias must be carefully considered [8,21,138,139,140]. Third, computational frameworks capable of integrating genomic, spatial, imaging, circulating, and clinical data will be required to translate complex biological measurements into interpretable clinical decisions. AI-based approaches offer considerable potential, but their clinical implementation will require rigorous external validation, transparency, robustness across institutions, and assessment of generalizability [142,143,144,145,147]. Clinical trial design will also need to evolve. Biomarker-driven and adaptive strategies may be better suited to evaluating treatment sequencing and ecosystem remodeling than conventional comparisons of fixed treatment regimens alone. Importantly, these approaches should continue to prioritize clinically meaningful outcomes, including event-free survival, OS, quality of life, and treatment-related toxicity, rather than relying exclusively on intermediate biological endpoints.

### 10.3. Conclusions

Breast cancer immunotherapy has achieved clinically established benefit in defined settings. In advanced TNBC, pembrolizumab plus chemotherapy is established for tumors with PD-L1 CPS ≥ 10, and sacituzumab govitecan plus pembrolizumab received FDA approval in June 2026 for the same biomarker-defined first-line population [75,76], and in high-risk early-stage TNBC, perioperative pembrolizumab plus chemotherapy improves pCR, event-free survival, and OS [10,11,18,31]. These findings establish ICB as a standard component of treatment for selected patients with TNBC. However, benefit remains heterogeneous, resistance is common, and the clinical roles of immunotherapy in HR+/HER2−, HER2-positive, and rarer breast cancer subtypes are not yet established [36,37,71,72,73,115,119]. The evidence reviewed here supports tumor microenvironmental plasticity as a useful framework for explaining this heterogeneity. Treatment response reflects interactions among malignant cells, stromal and vascular compartments, innate and adaptive immunity, and metabolic conditions, all of which may change during therapy [6,7,8,13,40,48]. Nevertheless, translating this biological framework into treatment selection remains an unmet goal. Beyond PD-L1 testing in advanced TNBC, multidimensional immune profiles, spatial biomarkers, circulating tumor DNA, and computational models have not yet been prospectively validated as routine tools for selecting immunotherapy combinations or guiding treatment sequence [4,21,147]. Likewise, although the success of perioperative immunotherapy demonstrates the importance of treatment context, the proposed immune advantages of specific schedules and biologically defined therapeutic windows remain promising but unproven [8,18,66].

Apart from established combinations in defined TNBC populations, strategies combining ICB with radiotherapy, endocrine therapy, CDK4/6 or DNA damage response inhibitors, other ADC regimens, and agents targeting myeloid, stromal, metabolic, or innate immune pathways are supported by mechanistic and early clinical evidence but should remain investigational until randomized trials demonstrate meaningful benefit and acceptable toxicity [16,48,58,119]. The immediate clinical priority is therefore to optimize evidence-based immunotherapy in populations with proven benefit. In parallel, prospective trials incorporating longitudinal sampling and standardized biomarker assessment should determine whether dynamic ecosystem models can be converted into reproducible clinical decision tools [8,140,147]. If validated, these approaches may enable rational patient selection and treatment sequencing and extend durable immune-mediated disease control to broader populations of patients with breast cancer.

## Figures and Tables

**Figure 1:**
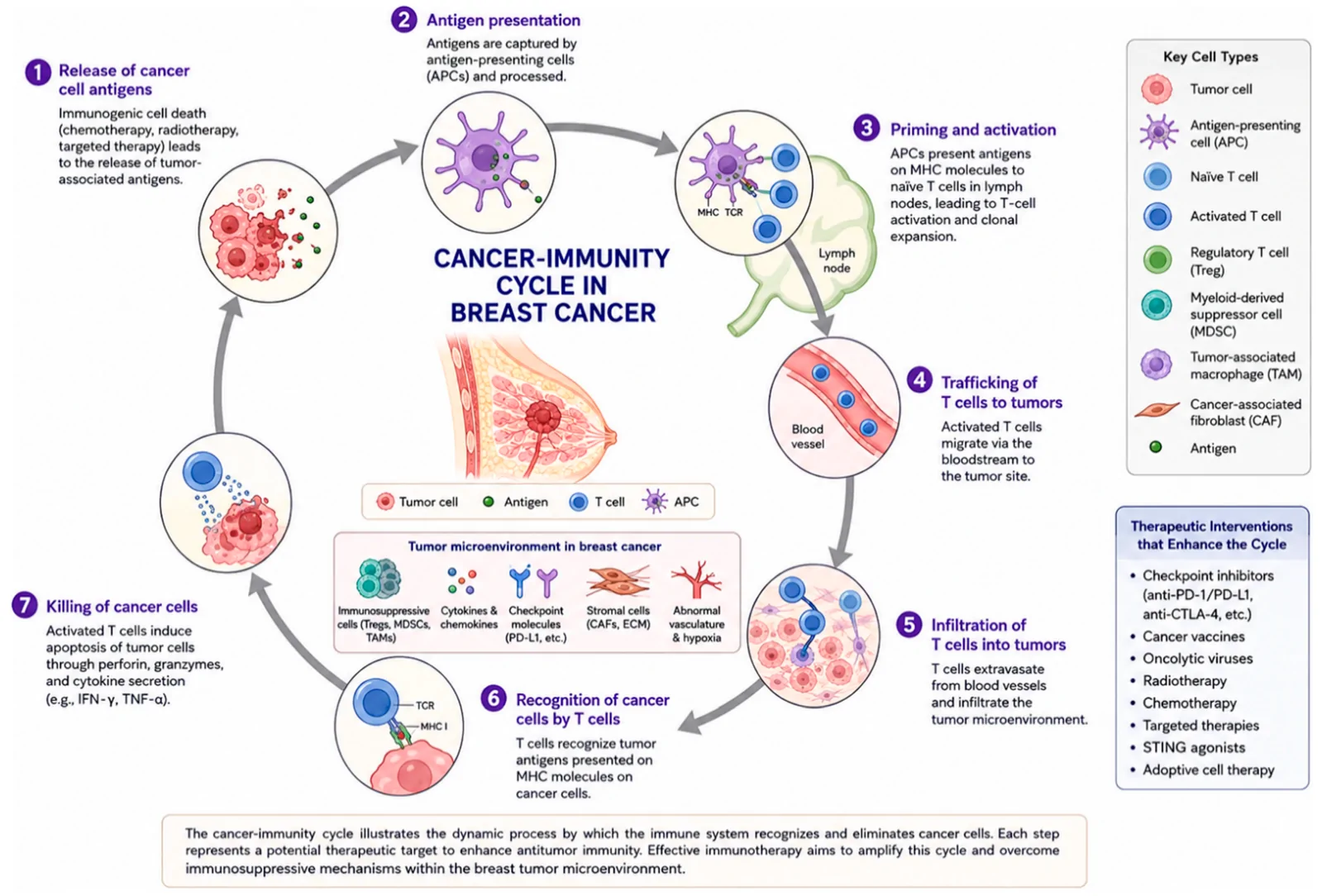
**Cancer-immunity cycle in breast cancer.** This schematic framework links antigen release, presentation, T-cell priming, trafficking, infiltration, recognition, and killing. Breast-cancer therapies may influence several steps, but the schematic framework does not establish efficacy of every illustrated intervention. APCs, antigen-presenting cells; DCs, dendritic cells; NK, natural killer; MDSCs, myeloid-derived suppressor cells; TAAs, tumor-associated antigens; ICI, immune checkpoint inhibitor.

**Figure 2:**
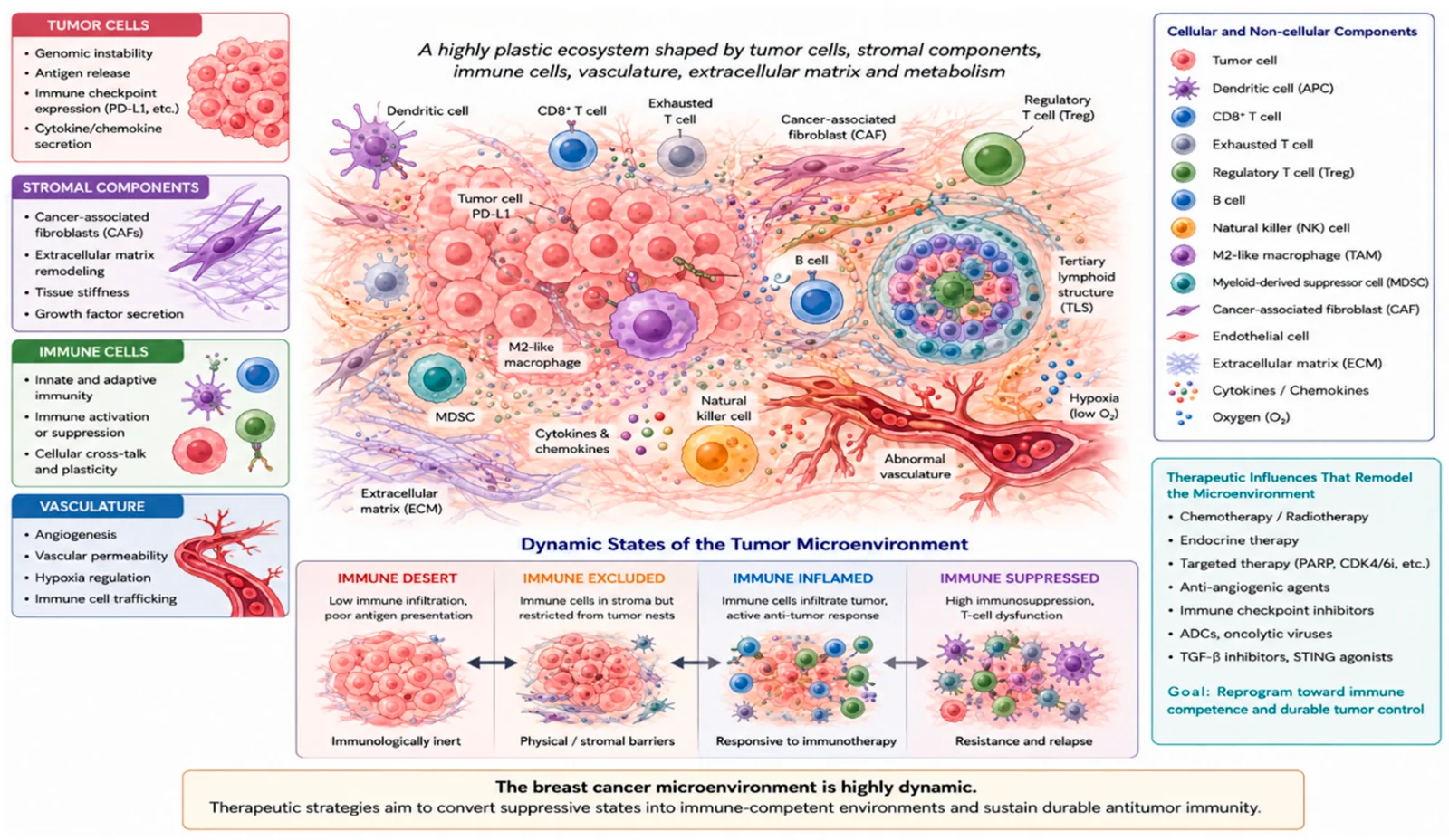
**Dynamic breast cancer microenvironment.** Cellular, stromal, vascular, and metabolic interactions influence immune function. Immune-desert, immune-excluded, and immune-inflamed denote spatial patterns; the illustrated suppressed state is a functional overlay that may accompany any pattern, not a fourth mutually exclusive spatial category. M1-like and M2-like labels denote simplified functional tendencies within a macrophage continuum, not discrete lineages. Therapy-induced changes are context-dependent and do not imply clinically validated conversion between states. Double-headed arrows indicate potential context-dependent bidirectional transitions between states and do not imply clinically validated conversion.

**Figure 3:**
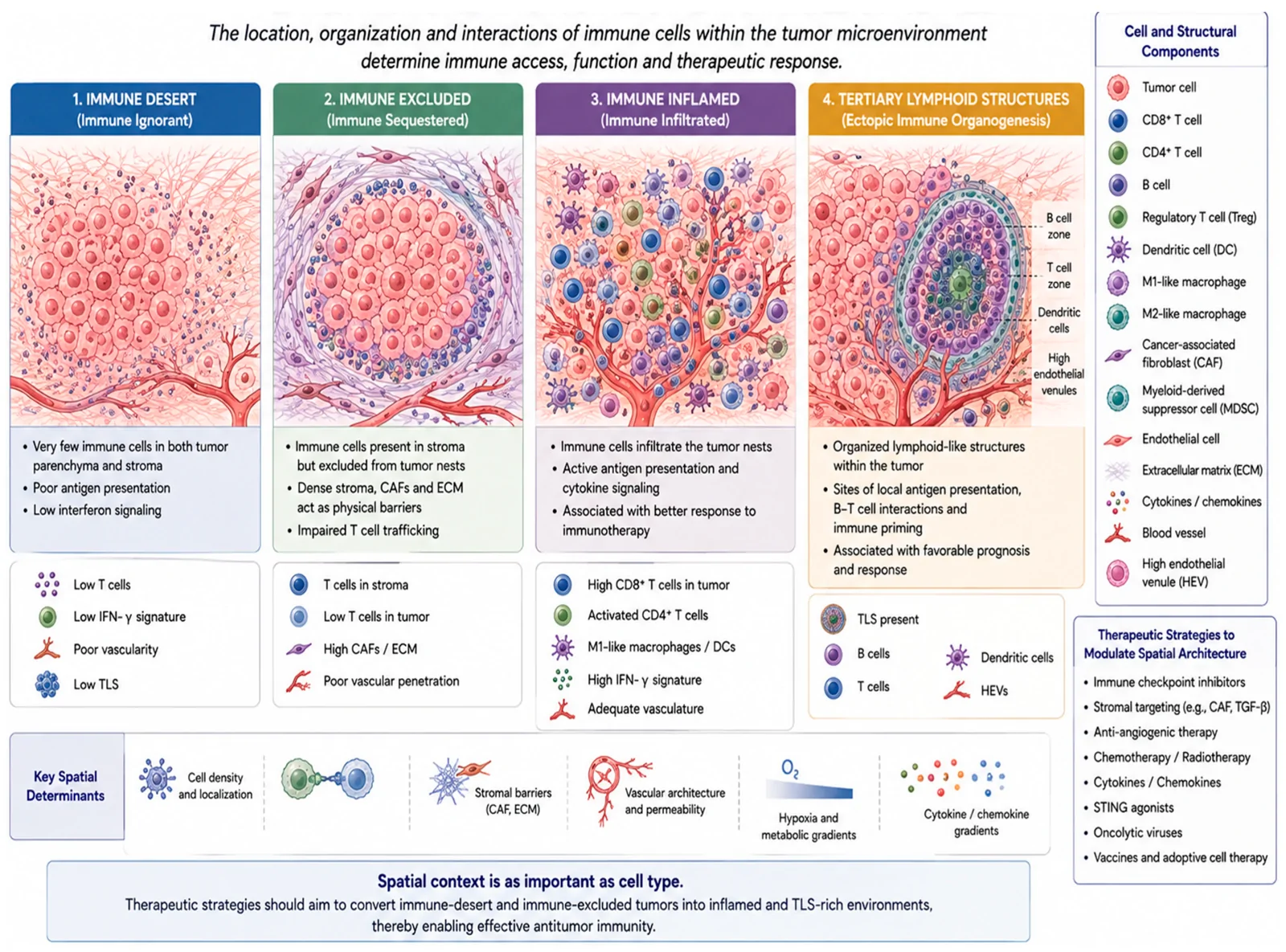
**Spatial immune architecture in breast cancer**. Immune-desert, immune-excluded, and immune-inflamed describe the location of immune cells relative to tumor tissue. TLS enrichment is an additional organizational feature, illustrated alongside these patterns rather than defined as a fourth mutually exclusive state. Different patterns and TLSs may coexist within a tumor. M1-like and M2-like macrophage labels are illustrative functional shorthand, not binary populations. TLS, tertiary lymphoid structure.

**Figure 4:**
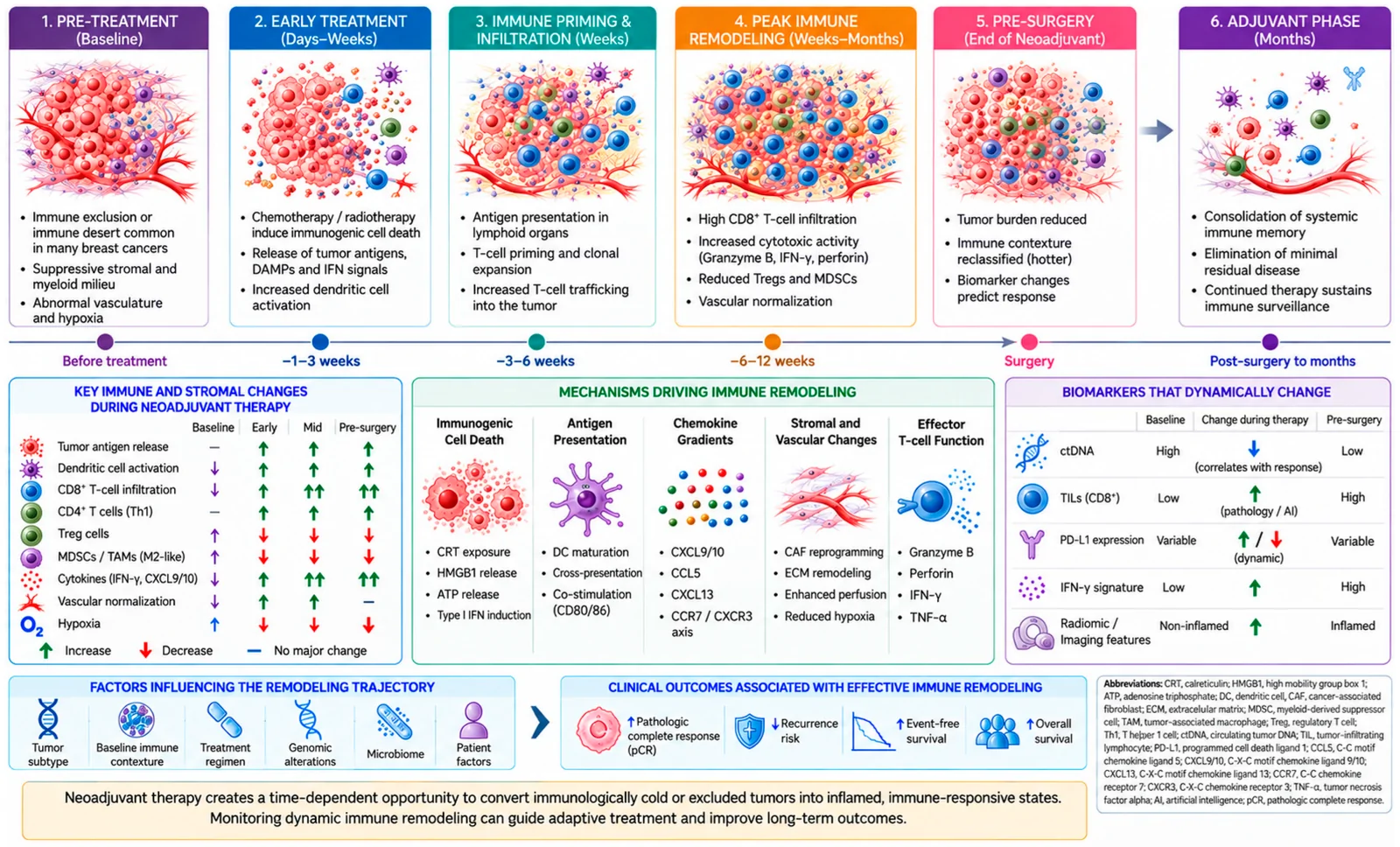
**Conceptual timeline of neoadjuvant immune remodeling in breast cancer**. Antigen release, priming, infiltration, and compensatory suppression may change during therapy. Serial tissue and blood measurements could test these transitions. The sequence and intervals are illustrative and do not define a validated sampling schedule or justify biomarker-directed treatment changes. TILs, tumor-infiltrating lymphocytes; ctDNA, circulating tumor DNA.

**Figure 5:**
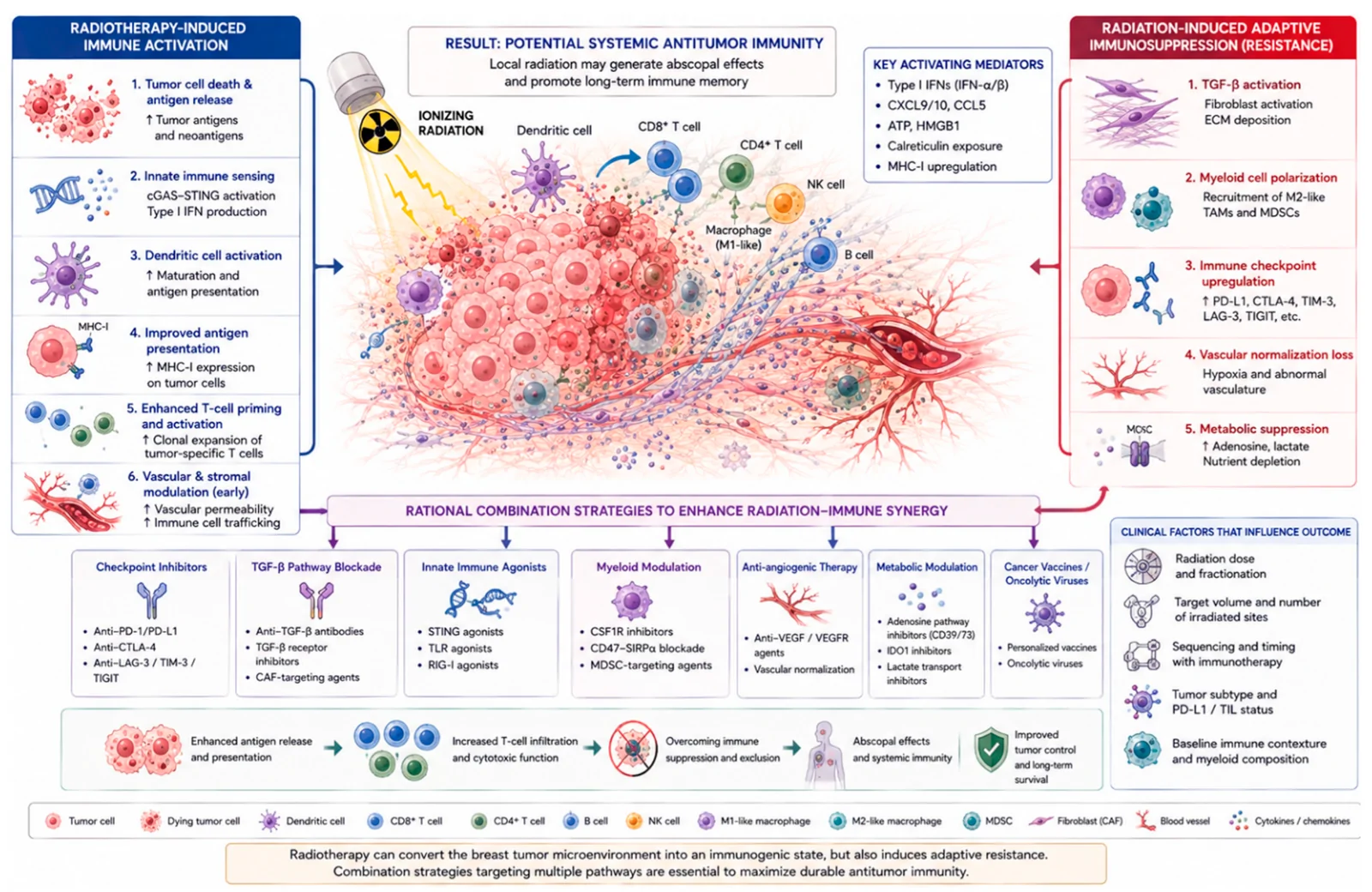
**Radiotherapy–immunotherapy crosstalk in breast cancer.** RT can promote antigen release and innate sensing while also causing lymphoid injury and activating stromal, vascular, myeloid, and metabolic suppression. Dose, fractionation, treated volume, and sequence influence these effects. Most mechanistic relationships remain preclinical or extrapolated from other cancers. M1-like and M2-like macrophage labels are functional shorthand along a continuum. RT-ICB combinations and abscopal responses should not be interpreted as established or reliably reproducible breast-cancer treatment effects.

**Figure 6:**
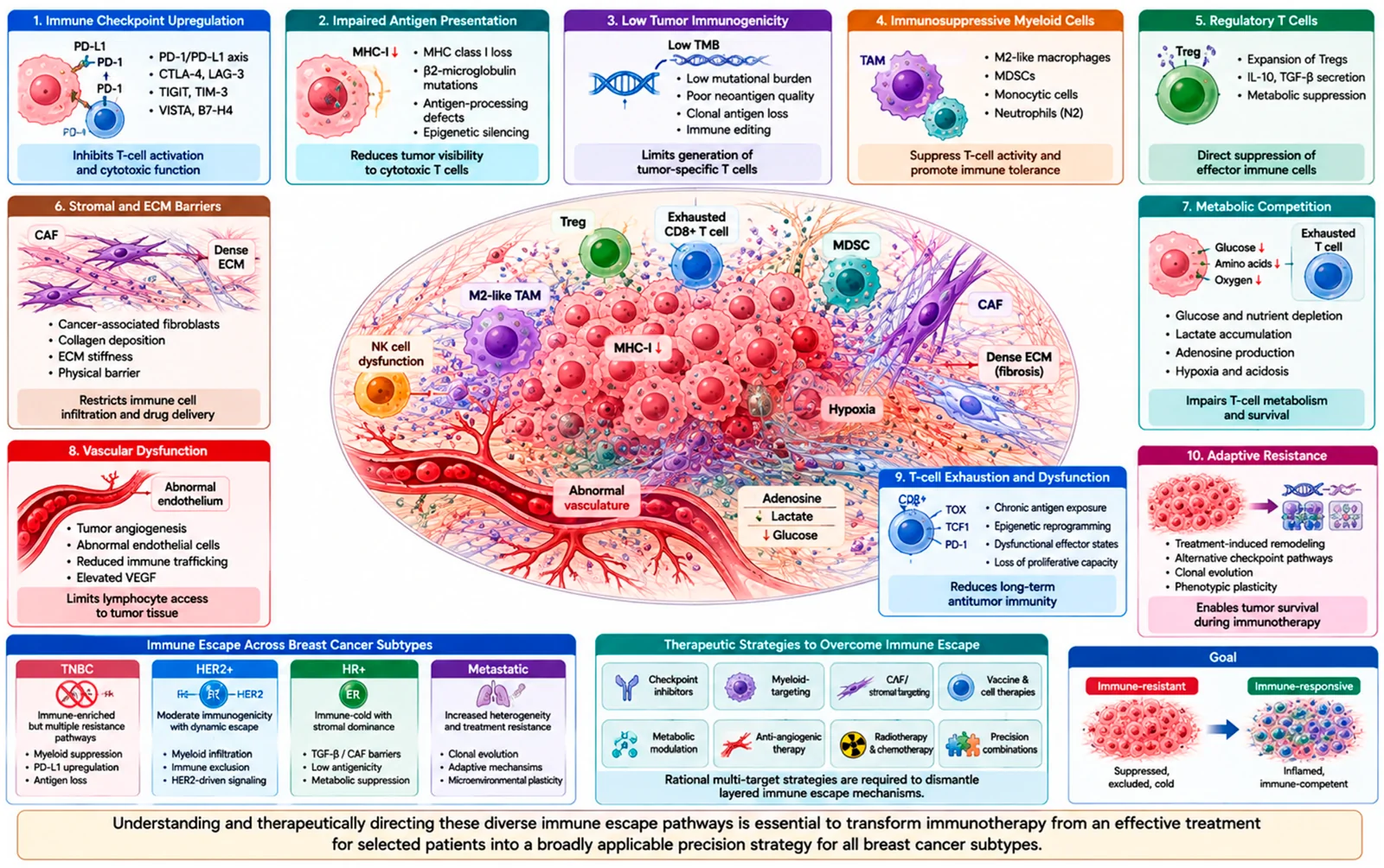
**Mechanisms of immune escape in breast cancer.** Tumor-intrinsic changes and immune, stromal, vascular, and metabolic constraints can coexist. Arrows indicate the direction of change (↑, increase; ↓, decrease) or transitions/interactions between cellular states and immune-escape mechanisms, as indicated. M1-like and M2-like macrophage labels indicate functional tendencies within a continuum. TNBC, HER2-positive, and HR-positive labels identify receptor-defined groups; metastatic identifies disease stage across those groups and is not a molecular subtype. The schematic organizes potential mechanisms and treatment contexts without asserting that each mechanism is clinically established in every breast-cancer setting.

**Figure 7:**
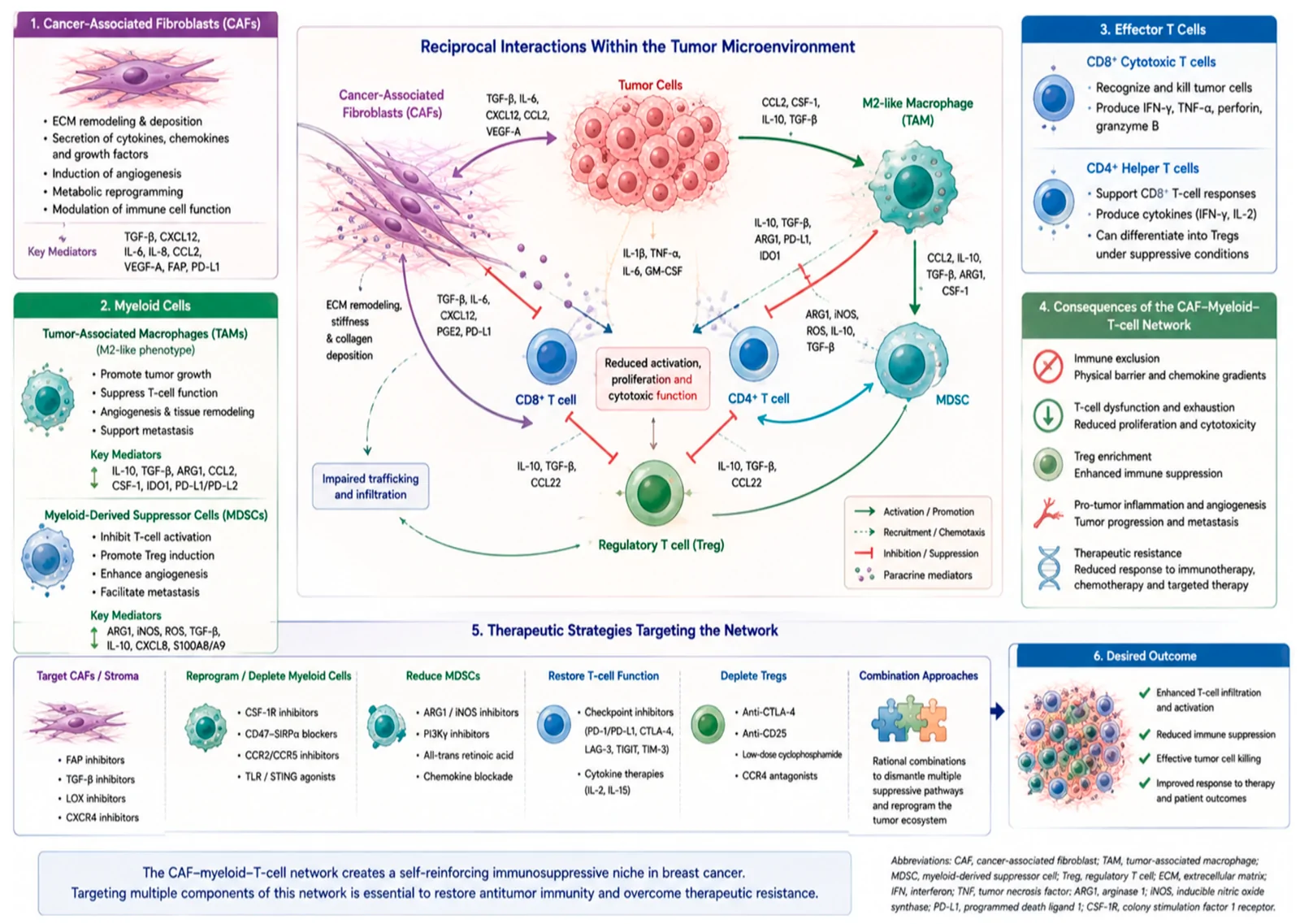
**CAF-myeloid-T cell network in the breast cancer microenvironment.** Stromal and myeloid signaling can restrict T-cell access and function. Arrows indicate the depicted interactions within the network: solid green arrows indicate activation or promotion, dashed green arrows indicate recruitment or chemotaxis, and red blunt-ended lines indicate inhibition or suppression; colored dots represent paracrine mediators. CAFs and macrophages are heterogeneous; M1-like and M2-like are illustrative functional tendencies rather than discrete or uniformly beneficial or harmful populations. The depicted interventions remain investigational in breast cancer. CAF, cancer-associated fibroblast; TAM, tumor-associated macrophage; MDSC, myeloid-derived suppressor cell.

**Figure 8:**
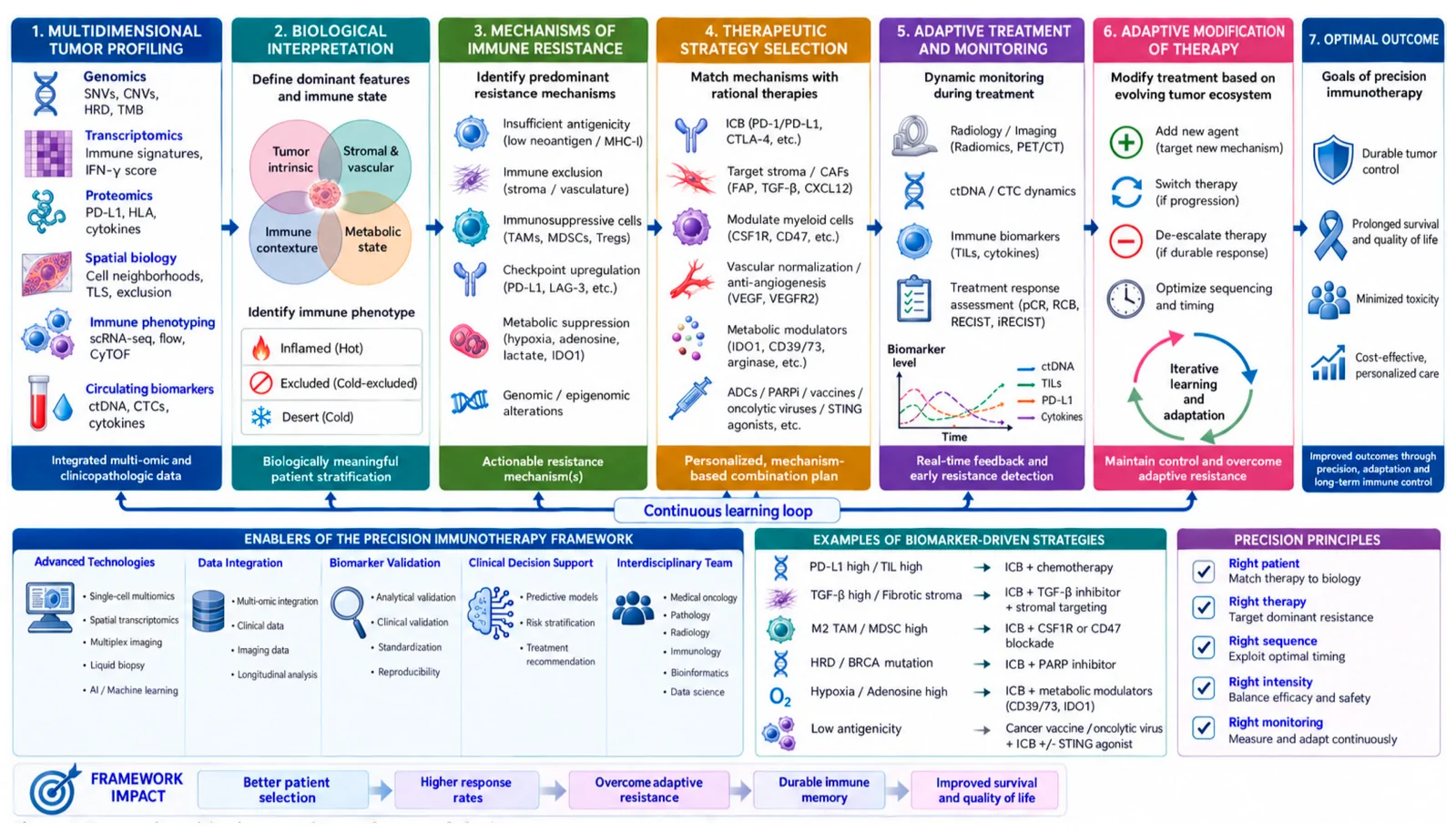
**Proposed precision immunotherapy framework for breast cancer**. Molecular, cellular, spatial, and circulating measurements may help characterize changing tumor states. The diagram is a research framework. Apart from established biomarkers in defined indications, multimodal profiling has not demonstrated routine clinical utility for selecting ICB combinations or directing escalation, de-escalation, or sequencing. Prospective interventional validation is required. Abbreviations: ADCs, antibody-drug conjugates; AI, artificial intelligence; BRCA, breast cancer gene; CAFs, cancer-associated fibroblasts; CD39, ectonucleoside triphosphate diphosphohydrolase 1; CD47, cluster of differentiation 47; CD73, ecto-5′-nucleotidase; CNVs, copy-number variations; CSF1R, colony-stimulating factor 1 receptor; CTCs, circulating tumor cells; CTLA-4, cytotoxic T-lymphocyte-associated protein 4; ctDNA, circulating tumor DNA; CXCL12, C-X-C motif chemokine ligand 12; CyTOF, cytometry by time of flight; FAP, fibroblast activation protein; HLA, human leukocyte antigen; HRD, homologous recombination deficiency; ICB, immune checkpoint blockade; IDO1, indoleamine 2,3-dioxygenase 1; IFN-γ, interferon gamma; LAG-3, lymphocyte-activation gene 3; M2, M2-polarized macrophage phenotype; MDSCs, myeloid-derived suppressor cells; MHC-I, major histocompatibility complex class I; PARPi, poly(ADP-ribose) polymerase inhibitor; pCR, pathological complete response; PD-1, programmed cell death protein 1; PD-L1, programmed death-ligand 1; PET/CT, positron emission tomography/computed tomography; RCB, residual cancer burden; RECIST, Response Evaluation Criteria in Solid Tumors; scRNA-seq, single-cell RNA sequencing; SNVs, single-nucleotide variants; STING, stimulator of interferon genes; TAMs, tumor-associated macrophages; TGF-β, transforming growth factor beta; TILs, tumor-infiltrating lymphocytes; TLS, tertiary lymphoid structures; TMB, tumor mutational burden; Tregs, regulatory T cells; VEGF, vascular endothelial growth factor; VEGFR2, vascular endothelial growth factor receptor 2.

**Table 1:** Landmark randomized trials and their Clinical interpretation.

Trial	Patient Population	Treatment	Endpoint(s)	Major Findings	Clinical Significance
KEYNOTE-355	Previously untreated metastatic TNBC; primary efficacy population with PD-L1 CPS ≥ 10	Pembrolizumab + investigator-choice chemotherapy vs. chemotherapy	PFS; OS	*n* = 847 randomized; CPS ≥ 10 subgroup OS 23.0 vs. 16.1 months; HR 0.73 (95% CI 0.55–0.95). Final OS analysis [10]	Established first-line pembrolizumab–chemotherapy for CPS ≥ 10 advanced TNBC [10].
IMpassion130	Previously untreated metastatic TNBC	Atezolizumab + nab-paclitaxel vs. nab-paclitaxel	PFS; OS	*n* = 902; final ITT OS 21.0 vs. 18.7 months; HR 0.87 (95% CI 0.75–1.02).	Activity with nab-paclitaxel does not establish interchangeability of chemotherapy backbones [27,28,29].
IMpassion131	Previously untreated metastatic TNBC	Atezolizumab + paclitaxel vs. paclitaxel	PFS; OS	*n* = 651; PD-L1-positive PFS 6.0 vs. 5.7 months; HR 0.82 (95% CI 0.60–1.12), *p* = 0.20. No OS benefit in the reported analysis [30].	Negative trial: no support for atezolizumab–paclitaxel in this setting [30].
KEYNOTE-522	Stage II–III early TNBC	Neoadjuvant pembrolizumab + chemotherapy followed by surgery and adjuvant pembrolizumab vs. chemotherapy	pCR; EFS; OS	n = 1174; 5-year OS 86.6% vs. 81.7%; HR 0.66 (95% CI 0.50–0.87); median follow-up 75.1 months [18].	Established perioperative regimen in eligible high-risk early TNBC [11,18,31].
IMpassion031	Early-stage TNBC	Neoadjuvant atezolizumab + chemotherapy followed by adjuvant atezolizumab vs. neoadjuvant placebo + chemotherapy	pCR; EFS	*n* = 333; pCR 58% vs. 41%; difference 17 percentage points (95% CI 6–27) [32] Final follow-up: EFS HR 0.76 (0.47–1.21), OS HR 0.56 (0.30–1.04) [33].	Investigational: pCR-positive; survival estimates imprecise and not independently definitive [32,33].
GeparNuevo	Early TNBC	Neoadjuvant durvalumab + chemotherapy vs. chemotherapy; short pre-chemotherapy immune-priming window	pCR; iDFS; DDFS; OS	*n* = 174. Primary endpoint: pCR 53.4% with durvalumab vs. 44.2% with placebo (OR 1.45; 95% CI 0.80–2.63; *p* = 0.287), not statistically significant. Long-term secondary outcomes at a median follow-up of 86.4 months: iDFS HR 0.56 (95% CI 0.32–0.99); OS HR 0.33 (95% CI 0.14–0.79).	Investigational phase II evidence: supports further study; does not establish a causal timing advantage [34].
NeoTRIPaPDL1	High-risk early/local TNBC	Neoadjuvant atezolizumab + carboplatin/nab-paclitaxel vs. chemotherapy	EFS primary; pCR secondary	*n* = 280; pCR 48.6% vs. 44.4%; OR 1.18 (95% CI 0.74–1.89), *p* = 0.48. This cited report concerns pCR, not definitive survival benefit [35].	Negative pCR result; no inference that timing alone explains differences from other trials [35].
KEYNOTE-756	High-risk, early-stage ER+/HER2− breast cancer	Neoadjuvant pembrolizumab + chemotherapy followed by surgery and adjuvant pembrolizumab + endocrine therapy vs. neoadjuvant placebo + the same chemotherapy followed by surgery and adjuvant placebo + endocrine therapy.	pCR; EFS	*n* = 1278; pCR 24.3% vs. 15.6%; estimated difference 8.5 percentage points (95% CI 4.2–12.8). EFS was immature	pCR improvement does not establish routine ICB use or a survival benefit in high-risk ER^+^/HER2^−^ disease [36].
CheckMate 7FL	High-risk, early-stage ER+/HER2− breast cancer	Neoadjuvant nivolumab + chemotherapy vs. placebo + chemotherapy, followed by surgery and adjuvant nivolumab + endocrine therapy vs. placebo + endocrine therapy; adjuvant placebo was subsequently removed by protocol amendment.	pCR; EFS	521 randomized; modified ITT *n* = 510 after exclusion of 11 patients. In the modified ITT population, pCR was 24.5% with nivolumab vs. 13.8% with placebo; difference 10.7 percentage points (95% CI 3.9–17.4). Survival interpretation is limited by protocol amendments and shortened follow-up [37].	Investigational: candidate subgroup associations do not establish a validated ICB-selection test [37].
A-BRAVE	High-risk early TNBC after surgery and (neo)adjuvant chemotherapy	One year of adjuvant avelumab vs. observation	DFS; OS	*n* = 477; median follow-up 52.1 months. ITT DFS HR 0.81 (95% CI 0.61–1.09), *p* = 0.172. Descriptive OS signal; primary DFS endpoints not met [38].	Negative primary endpoint: does not establish routine adjuvant avelumab or prove inferiority of postoperative timing [38].

Table note: Values compare experimental with control treatment within each trial. n is the randomized total unless specified; effects refer to the stated analysis population. CI, confidence interval; HR, hazard ratio; OR, odds ratio; ITT, intention to treat; pCR, pathological complete response; EFS, event-free survival; iDFS, invasive disease-free survival; DDFS, distant disease-free survival; OS, overall survival; DFS, disease-free survival; PFS, progression-free survival; CPS, combined positive score. Survival maturity refers to the cited report. Differences in design, population, treatment, endpoints, and follow-up preclude efficacy ranking or causal timing inference across trials.

**Table 2:** Cellular and Non-Cellular Components of the Breast TME.

Component	Major Functions	Effect on Antitumor Immunity	References
Tumor cells	Antigen presentation, cytokine/chemokine production, checkpoint-ligand expression, metabolic competition	Can initiate immune activation but also drive immune escape and immunoediting	[6,7,26]
CD8+ T cells	Direct cytotoxic tumor-cell killing	Major mediators of effective adaptive antitumor immunity	[8,42,43,44]
CD4+ helper T cells	Cytokine production; coordination of T-, B-, and myeloid-cell responses	Support immune activation and memory formation	[8,13,41]
Regulatory T cells (Tregs)	Immune suppression and regulation of effector responses	Can restrict cytotoxic T-cell and antigen-presenting-cell activity	[8,13,41]
B cells	Antigen presentation, antibody production, cytokine signaling	Can support antitumor immunity and TLS formation; effects are context-dependent	[19,20,45]
Dendritic cells	Antigen capture, processing, and cross-presentation	Essential for initiation of tumor-specific adaptive immunity	[46]
Natural killer cells	Innate cytotoxicity and recognition of stressed/transformed cells	Contribute to elimination of susceptible tumor cells and immune surveillance	[47]
Tumor-associated macrophages	Tissue remodeling, angiogenesis, cytokine production, phagocytosis	Can promote or suppress immunity according to functional state and context	[48,49]
Myeloid-derived suppressor cells (MDSCs)	Suppression of T-cell and NK-cell function; metabolic and inflammatory regulation	Promote immune escape and therapeutic resistance	[50,51]
Neutrophils	Inflammation, ECM remodeling, cytokine signaling	Highly context-dependent; can exert either tumor-promoting or tumor-restraining effects	[48,50]
Cancer-associated fibroblasts	ECM deposition, collagen remodeling, chemokine/cytokine production	Can promote immune exclusion and immunosuppressive niches; functions are heterogeneous	[52,53,54]
Endothelial cells/tumor vasculature	Angiogenesis, oxygen delivery, leukocyte adhesion and trafficking	Regulate immune-cell access to tumor tissue	[55,56]
Extracellular matrix	Structural support, mechanical signaling, growth-factor sequestration	Dense or remodeled ECM can restrict immune-cell migration and drug delivery	[12,40,52]
Cytokines and chemokines	Intercellular signaling and recruitment	Can support immune activation or establish suppressive cellular niches depending on context	[13,40,41]
Hypoxia and metabolic factors	Regulate nutrient availability, oxygenation, lactate and adenosine signaling	Can impair T-cell/NK-cell function and promote immunosuppressive cell states	[57,58,59,60]

Table note: The TME should not be interpreted as a collection of uniformly pro- or anti-tumor cell types. Most components exhibit substantial functional plasticity depending on tumor location, disease stage, metabolic conditions, and treatment exposure.

**Table 3:** Additional Phase III Trials in Breast Cancer Immunotherapy.

Trial/Program	Disease Setting	Treatment Strategy	Current Evidence/Status	Scientific Rationale
SWOG S1418/NRG BR006	TNBC with residual invasive disease after neoadjuvant chemotherapy	Adjuvant pembrolizumab vs. observation	Phase III study closed to accrual (SWOG S1418/BR006 trial record). Trial design described in the cited protocol report; no efficacy result is asserted here [74].	Tests whether postoperative checkpoint blockade benefits patients with residual high-risk disease after neoadjuvant chemotherapy [74].
ASCENT-04/KEYNOTE-D19	Previously untreated PD-L1-positive unresectable locally advanced/metastatic TNBC	Sacituzumab govitecan + pembrolizumab vs. chemotherapy + pembrolizumab	Phase III; n = 443. Median PFS was 11.2 months with sacituzumab govitecan + pembrolizumab vs. 7.8 months with chemotherapy + pembrolizumab (HR 0.65; 95% CI 0.51–0.84; *p* = 0.0009) [75]. OS was immature in the analysis summarized in the FDA’s 24 June 2026 approval notice [76]. The combination was approved on that date for first-line treatment of adults with unresectable locally advanced or metastatic TNBC with PD-L1 CPS ≥ 10, determined by an FDA-authorized test.	Demonstrates PFS benefit for this specific ADC-ICB regimen, not a class-wide advantage [75].
Platform and biomarker-driven trials	Multiple breast cancer subtypes	ADCs, DDR inhibitors, CDK4/6 inhibitors, radiotherapy, innate immune agonists, and next-generation checkpoint combinations	Active area of clinical investigation	Seeks to match treatment combinations to specific mechanisms of immune resistance rather than applying universal combinations [14,16,17,64,75,77].

**Table 4:** Emerging Therapeutic Targets Beyond PD-1/PD-L1.

Target/Pathway	Major Biological Function	Development Status/Strategy	Principal Rationale	References
CTLA-4	T-cell priming and regulation	Established checkpoint target in other cancers; combinations under investigation in breast cancer	Enhance T-cell priming and broaden antitumor responses	[1,2,85,92]
LAG-3	T-cell dysfunction/exhaustion	LAG-3-directed checkpoint combinations under clinical investigation	Complement PD-1 pathway inhibition	[77,111,112,113]
TIGIT	T-cell and NK-cell inhibitory signaling	Investigational checkpoint combinations	Restore lymphocyte effector function	[47,77]
TIM-3	Dysfunctional/terminal immune states	Investigational checkpoint blockade	Target complementary exhaustion pathways	[77,111,112]
VISTA	Myeloid and T-cell immune suppression	Early clinical/investigational development	Reprogram suppressive immune niches	[109]
TGF-β	Fibrosis, immune exclusion and immune suppression	Investigational TGF-β inhibitors/antibodies	Improve T-cell infiltration and overcome stromal exclusion	[23,80]
STING	Cytosolic DNA sensing and type I interferon signaling	STING agonists in clinical investigation	Activate innate sensing and enhance antigen presentation	[64,88]
CD47-SIRPα	Macrophage phagocytic checkpoint	Anti-CD47/SIRPα strategies under investigation	Enhance macrophage-mediated tumor-cell clearance	[106,110]
CSF1/CSF1R	Macrophage recruitment and differentiation	Macrophage-targeted agents in clinical investigation	Reprogram or reduce suppressive myeloid populations	[48,49]
CXCR4/CXCL12	Stromal signaling and immune exclusion	Investigational chemokine-pathway inhibitors	Improve immune-cell trafficking into tumor tissue	[51]
FAP	CAF-associated stromal remodeling	FAP-directed antibodies, radioligands, vaccines, and cellular approaches under investigation	Target tumor-promoting stromal compartments	[52,54]
VEGF/VEGFR	Angiogenesis and vascular dysfunction	Anti-angiogenic therapy; established in selected breast-cancer contexts but combinations remain context-dependent	Improve vascular function and immune-cell trafficking	[55,56]
IDO1	Tryptophan metabolism and immune suppression	Investigational; phase III epacadostat–pembrolizumab failed to improve outcomes in melanoma [114].	Restore metabolic conditions favorable for T-cell function	[114]
CD39/CD73-adenosine	Extracellular adenosine production	Investigational inhibitors	Reduce adenosine-mediated immune suppression	[60]
Metabolic/hypoxia pathways	Nutrient competition and immune dysfunction	Experimental/early clinical development	Restore metabolic fitness of immune cells	[57,58,59]

**Table 5:** Mechanistic Basis for Rational Combination Immunotherapy.

Combination Strategy	Expected Immunological Effect	Evidence/Clinical Status
ICB + chemotherapy	Increased antigen availability, dendritic-cell activation, and T-cell priming	Clinically established in selected TNBC settings; particularly strong evidence from KEYNOTE-522 and KEYNOTE-355 [10,31]
ICB + radiotherapy	Increased inflammatory signaling, antigen presentation, and potentially systemic immunity	Investigational in breast cancer; regimen, dose, fractionation, and sequencing remain unresolved [16,64,65,94,95,96,128,129]
ICB + endocrine therapy	Potential enhancement of immune accessibility in selected HR+ tumors	Investigational; standalone benefit from endocrine therapy plus ICB is not established by the evidence summarized here. KEYNOTE-756 evaluated a broader perioperative regimen incorporating neoadjuvant pembrolizumab plus chemotherapy followed by adjuvant pembrolizumab plus endocrine therapy; it does not isolate the contribution of the endocrine-ICB combination (see Table 1) [36]
ICB + CDK4/6 inhibition	Enhanced immune activation and cytotoxic T-cell function	Investigational; hepatic and pulmonary toxicity limits some CDK4/6–ICB combinations [14,116].
ICB + PARP inhibition	Increased interferon signaling and tumor immunogenicity	Investigational; established PARP activity in BRCA-mutated disease does not establish combination efficacy [17,117,118]
ICB + ADCs	Enhanced tumor-cell killing and immune stimulation	One established US regimen: sacituzumab govitecan + pembrolizumab for first-line CPS ≥10 advanced TNBC. Other ADC–ICB regimens require separate evidence [75].
ICB + anti-angiogenic therapy	Enhanced lymphocyte access to tumor tissue	Investigational in breast cancer; biological rationale supports combining anti-angiogenic therapy with ICB, but the cited evidence does not establish clinical benefit or support routine use of this combination in breast cancer [55,56]
ICB + TGF-β inhibition	Enhanced T-cell infiltration and reduced stromal suppression	Investigational; strong mechanistic rationale but breast-cancer-specific clinical validation remains limited [23,80]
ICB + STING agonists	Enhanced dendritic-cell maturation and T-cell priming	Investigational; delivery and toxicity remain major considerations [64,88]
ICB + cancer vaccines	Expansion of tumor-reactive T cells and potential immune memory	Investigational; personalized vaccines remain an active clinical research area [127]
ICB + oncolytic viruses	Enhanced antigen release, innate activation, and T-cell priming	Investigational with clinically validated activity in selected cancers; breast-cancer efficacy remains under study [126]
Multi-modal precision combinations	Coordinated remodeling of the tumor ecosystem	Emerging research paradigm requiring biomarker-driven prospective validation

## Data Availability

No new data were created or analyzed in this study. Data sharing is not applicable to this article.
